# Ab initio generalized Langevin equation

**DOI:** 10.1073/pnas.2308668121

**Published:** 2024-03-29

**Authors:** Pinchen Xie, Roberto Car, Weinan E

**Affiliations:** ^a^Program in Applied and Computational Mathematics, Princeton University, Princeton, NJ 08544; ^b^Department of Chemistry and Princeton Materials Institute, Princeton University, Princeton, NJ 08544; ^c^Department of Physics, Princeton University, Princeton, NJ 08544; ^d^AI for Science Institute, Beijing 100080, China; ^e^Center for Machine Learning Research and School of Mathematical Sciences, Peking University, Beijing 100084, China

**Keywords:** multiscale modeling, generalized Langevin equation, machine learning

## Abstract

We introduce a machine learning–based approach called ab initio generalized Langevin equation (AIGLE) to model the dynamics of slow collective variables (CVs) in materials and molecules. In this scheme, the parameters are learned from atomistic simulations based on ab initio quantum mechanical models. Force field, memory kernel, and noise generator are constructed in the context of the Mori–Zwanzig formalism, under the constraint of the fluctuation–dissipation theorem. Combined with deep potential molecular dynamics and electronic density functional theory, this approach opens the way to multiscale modeling in a variety of situations. Here, we demonstrate this capability with a study of two mesoscale processes in crystalline lead titanate, namely the field-driven dynamics of a planar ferroelectric domain wall, and the dynamics of an extensive lattice of coarse-grained electric dipoles. In the first case, AIGLE extends the reach of ab initio simulations to a regime of noise-driven motions not accessible to molecular dynamics. In the second case, AIGLE deals with an extensive set of CVs by adopting a local approximation for the memory kernel and retaining only short-range noise correlations. The scheme is computationally more efficient than molecular dynamics by several orders of magnitude and mimics the microscopic dynamics at low frequencies where it reproduces accurately the dominant far-infrared absorption frequency.

## Significance

Recent developments of neural network–based atomistic models enable large scale molecular dynamics based on quantum mechanical theory. However, there is still a gap between modeling microscale molecular dynamics and modeling mesoscale coarse-grained (CG) dynamics with the same level of accuracy. The ab initio generalized Langevin equation (AIGLE) model is proposed to bridge the gap, by learning accurately an effective equation of motion for multidimensional CG variables from molecular dynamics data. The capability of AIGLE is demonstrated by simulating domain wall dynamics and extensive dipolar motion in the ferroelectric crystal lead titanate. This work is a step toward bottom–up modeling of mesoscale transformations in materials.

Developing accurate and reliable mesoscale physical models is a long-standing problem (1–3). In this context, the Mori–Zwanzig formalism (4) stands out as a general methodology for constructing effective coarse-grained (CG) models for any set of collective variables (CVs) defined in terms of microscopic degrees of freedom, such as the atomic coordinates. The idea is to project the dynamics of the microscopic variables on the space of the CVs. Finding an approximate surrogate model for the formal projective dynamics requires knowledge of the free energy as a function of the CVs, and brings in two important effects: memory, because CV dynamics is generally non-Markovian, and noise, associated with the initial condition for the variables eliminated in the projection process. These effects are difficult to model. As a consequence, one often resorts to simpler approximations for the effective dynamics, such as the Markovian Langevin equation (LE).

Combined with Landau free energy models (5–7), LE has been a popular tool for describing mesoscale dynamical processes. Well-known examples include the Landau–Lifshitz equation for the evolution of the magnetization in materials (8), the Allen-Cahn and the Cahn–Hilliard equations for the dynamics of phase transitions and separations (9, 10), and, more generally, the phase field models (11) and the phase-field-crystal models (12, 13) for a variety of problems in materials science. Landau-based LEs provide invaluable physical insight but may lack the flexibility required to quantitatively model the CG dynamics of real systems. A main issue is the insufficient separation of time scales between the CVs and the noise. In realistic systems, noise may originate from vibrational modes that are not significantly faster than the CVs. In this scenario, the non-Markovian generalized Langevin equation (GLE) is a much better approximation. It can be rigorously derived within the Mori–Zwanzig formalism for Hamiltonians that depend quadratically on the microscopic degrees of freedom (4). In the presence of anharmonicity, the GLE is not exact but can be a flexible enough tool for connecting micro- and mesoscale dynamics, similar in spirit to the way in which semilocal density functional theory (DFT) bridges electronic quantum mechanics and atomistic models (14). So far, efforts to develop quantitatively accurate GLE models have been limited by difficulties in the parameterization of the memory and noise terms (15–17). In the context of bottom–up multiscale modeling, these difficulties lie in the lack of microscopic data, on the one hand, and of robust algorithms to parameterize the GLE, on the other.

In recent years, machine learning has emerged as a powerful tool in the study of static and dynamic statistical properties of molecular systems (18–23), enabling ab initio simulations of unprecedented scale (25, 26). Today, massive amounts of data can be generated by all-atom molecular dynamics (MD) trajectories with ab initio accuracy. As we will demonstrate below, machine learning can also address the second difficulty mentioned above.

In this paper, we introduce a machine learning–based method for constructing accurate CG GLE models from fine-grained/microscopic Hamiltonians. We illustrate the approach with atomistic models derived from DFT, but the methodology can also be applied to microscopic models derived phenomenologically. In our scheme, memory is of finite length and translationally invariant in time, and the noise satisfies the constraint imposed by the second fluctuation–dissipation theorem (2FDT) (27), which connects the memory kernel to the autocorrelation function (ACF) of the noise. The 2FDT is essential to describe the dynamics of near-equilibrium physical systems. We call the schemes constructed in this way ab initio generalized Langevin equation (AIGLE) models, because they are trained on data generated with an ab initio microscopic model. The LE model derived from AIGLE by taking the Markovian limit in the memory kernel and the noise will be called ab initio Langevin equation (AILE) model.

Previous works have studied data-driven parameterizations of the GLE (28–43), the LE (44–46), the far-from-equilibrium GLE (47, 48), and generic stochastic processes (49, 50). For GLE restricted by 2FDT, the noise generator is usually constructed from a predetermined memory kernel or from the ACF of the noise. For instance, in refs. 29 and 30, the noise generator is a Yule–Walker linear autoregressive (AR) model fitted to the ACF of the noise. The resulting model does not guarantee the stationarity of the noise. That can be imposed by adjusting the roots of the characteristic equation, but this may lead to uncontrolled errors. Another approach, reported in refs. 31 and 32, assumes that the noise generator is a Fourier series with random coefficients sampled from a distribution defined by the memory kernel. In practice, the Fourier series is of finite length, and the generated noise and its ACF become periodic. Recently, ref. 41 proposes to convert a GLE into Markovian equations of motion for fictitious degrees of freedom, by using a finite order Padé approximant for the memory kernel. The dynamics of the fictitious degrees of freedom is constructed according to the memory kernel while retaining the 2FDT constraint on the noise. In general, approaches that use an average property like the ACF to fix the noise are “mean-field” approximations, aiming at consistency with data on 2FDT rather than on presumably less relevant features like higher-order correlations or kurtosis. Concerns have been raised that in some of these approaches, the statistical error of the correlation functions may be amplified in an uncontrolled way (43).

Alternatively, one can go beyond “mean-field” by adopting a regression approach. For example, ref. 49 introduced a nonlinear autoregressive model for generic stochastic processes not constrained by the 2FDT. Refs. 50 and 51 used recurrent neural networks for learning generic dynamical systems, as the nonlinear nature of general regression tasks may require sophisticated deep neural network models. However, in specialized but important cases such as near-equilibrium systems, knowledge of the free energy surface (FES) and the 2FDT facilitate the task, making it possible to reproduce the time series with relatively shallow and simple neural network regressors. Then, accuracy, stationarity, and efficiency can be achieved simultaneously. In AIGLE, we strive to optimize these three qualities, while “mean-field” approaches essentially compromise accuracy. We constrain the memory kernel with the 2FDT and model the noise with a neural network-based generalized autoregressive (GAR) scheme that can deal with insufficient time-scale separation and anharmonic coupling of the modes. These complications are common in real materials but are often overlooked in toy models. To have an efficient noise generator suited for long-time simulation, we keep the neural network as simple as a compact feed-forward neural network. While most data-driven GLEs assume prior knowledge of the FES, in AIGLE not only the noise but also the FES and the couplings to the driving fields can be parameterized. Moreover, while most existing literature uses a one-dimensional GLE, we introduce a multidimensional version of AIGLE, based on a local kernel approximation and a consistent GAR model, which can reproduce not only the one-body but also local two-body correlations. The adopted approximation balances efficiency and accuracy, making it possible to deal with infinite-dimensional, homogeneous CVs. To our knowledge, multidimensional GLEs have only been used so far to study the few-body dynamics of low-dimensional CVs (52–54).

In this paper, we expose the details of AIGLE and demonstrate its effectiveness in an ab initio multiscale study of ferroelectric lead titanate (PbTi$\;{\text{O}}_{3}$). The scheme is not limited to ferroelectric problems and its mathematical structure can be used in reduced models of general crystalline materials. In the present application, the order parameters, i.e., the CVs, depend on the local electric dipole moments associated with the crystalline lattice. These local moments act like lattice spins in ferromagnets, and, as the latter, can be coarse grained to scalar or vector fields in the continuum limit. Unlike lattice spins of fixed magnitude, the local dipoles fluctuate in both direction and magnitude. The sum of the local dipoles defines the polarization of the system, which is a typical example of a global order parameter in Landau’s theory of symmetry breaking. Damped vibrational modes associated with polar phonons are embedded in the dynamics of the local dipoles, inducing oscillating correlations among the dipoles, a behavior that differs significantly from the diffusive dynamics of Brownian particles, whose velocity ACF decays exponentially with time. Hence, the difficulties encountered in CG dipole dynamics are similar to those encountered in realistic multiscale models of materials and macromolecules.

Specifically, we consider two examples of mesoscale dynamics in crystalline materials: the field-driven dynamics of a planar ferroelectric domain wall treated as a virtual particle in a non-Markovian bath and the dynamics of extensive local order parameters with translationally invariant interactions. In both cases, AIGLE is trained with atomic trajectories generated at room temperature with the Deep Potential (DP) scheme (21), a deep learning approach that closely reproduces the quantum mechanical potential energy surface at the DFT level of theory. The microscopic lattice dipoles, rigorously defined in the theory of the electric polarization within DFT (55, 56), are represented by an equivariant generalization of the DP model (57). In the first example, we study the glassy dynamics of a planar 180° domain wall induced by a weak electric field $E=E\hat{z}$ parallel to the polarization of one of the domains. We find that the domain wall shifts by a succession of rare events. At low fields, the domain velocity $v_{D}$ predicted by AIGLE gradually deviates from its AILE counterpart and from the phenomenological scaling law of Merz (58), according to which $v_{D}\propto e^{-E_{a}/E}$, with constant $E_{a}$. Merz’s law can be derived from the theory of elastic interface motion (59, 60) that describes the dynamics with an overdamped Langevin equation. Our results suggest that inertia and memory effects captured by AIGLE play a role in the glassy motion of elastic interfaces under weak applied fields. In the second example, we consider the dynamics of a CG lattice of dipoles in the bulk of a compressively strained PbTi$\;{\text{O}}_{3}$ crystal. AIGLE reproduces well self- and close-neighbor correlations of the dipoles and captures approximately the ACF of the time derivative of the polarization, whose Fourier transform yields the far-infrared optical spectrum. AILE fails in this task but still models correctly the relaxation pattern of a domain structure, when this is driven by surface tension and memory is not important. A CG lattice dynamics of extensive CVs like that provided here by AIGLE or AILE would be useful, in general, in studies of the dynamics of extended crystal defects and of epitaxial growth of materials.

The paper is organized as follows. In Section 1, we introduce the AIGLE formalism. In Section 2, we use AIGLE for ab initio multiscale modeling of PbTi${\text{O}}_{3}$. Specifically, we report in Section A a model for the field-driven motion of a planar domain wall in epitaxial PbTi${\text{O}}_{3}$. In Section B, we report an extensive model of CG lattice dynamics. Details of one-dimensional AIGLE are in the Material and Methods section. Details of multidimensional AIGLE are in *SI Appendix*, which also includes the microscopic models for PbTi${\text{O}}_{3}$ and other technical details.

## 1. The AIGLE Model

The starting point is a microscopic model of molecular dynamics. CVs, obtained by coarse-graining the microscopic degrees of freedom when constructing the FES, form a column vector x. The aim is to eliminate the remaining degrees of freedom, and obtain an accurate dynamic model for the CVs, using the GLE ansatz:

[1] $$\begin{array}{c} M\frac{d^{2}x\left(t\right)}{dt^{2}}=-\nabla G\left(x\right)+F\left(t\right)+\int_{0}^{t}dsMK\left(s\right)\frac{dx(t-s)}{\mathrm{dt}}+R\left(t\right). \end{array}$$

Here, M is the effective mass matrix, G(x) is the FES, the vector F comprises the external driving forces, K is the memory kernel matrix, and the vector R represents the noise. We define $v=\frac{dx}{\mathrm{dt}}$, $a=\frac{d^{2}x}{dt^{2}}$, F=−∇G(x)+F, and use the subscript T for the transpose of a vector or a matrix. We shall use the brackets ⟨⋯⟩ to indicate an average over the equilibrium ensemble at t=0. We require ⟨R(t)⟩=0, and the orthogonality condition $\langle R\left(t\right)v^{T}\left(0\right)\rangle =0$, from which the 2FDT can be derived (27). The 2FDT prescribes that, at equilibrium, $\left\langle v\left(0\right)v^{T}\left(0\right)\right\rangle K^{T}\left(s\right)=-\left\langle \left(M^{-1}R\right)\left(0\right){\left(M^{-1}R\right)}^{T}\left(s\right)\right\rangle$, relating the memory kernel to the ACF of the noise. In addition, although the noise should not be strictly stationary, $\left\langle R\left(t_{0}+t\right)R^{T}\left(t_{0}\right)\right\rangle$ should be independent of $t_{0}$ for sufficiently large $t_{0}$.

In AIGLE, Eq. **1** is learned from the trajectories of x. The scheme can use, but does not require, a predetermined FES (61–69), as all the terms in Eq. **1** can be learned from adequate trajectory data. The resulting GLE satisfies numerically the 2FDT for the equilibrium ensemble, and the model can be extended to near-equilibrium dynamics. Extensions to general nonlinear dynamics beyond the 2FDT would be possible, but this paper is limited to near-equilibrium situations. We show that the scheme can be constructed from large-scale MD simulations of realistic materials models. First, we formulate AIGLE for a one-dimensional CV and then we generalize it to infinite-dimensional lattice CVs.

To learn from time series data generated by MD, it is convenient to transform the integro-differential equation [**1**] into discrete form. We assume that the memory kernel is homogeneous, i.e., independent of position (70) and time origin (71), and use Δt for the time step of the discretized GLE. Setting t=0 for the arbitrary starting time, the current time is t=nΔt, and we use the notation $f_{\left(n\right)}$ to indicate a time-dependent function f(nΔt). Then, the discretized form of Eq. **1** for a one-dimensional CV reads

[2] $$\begin{array}{c} {ma}_{\left(n\right)}=-\nabla G\left(x_{\left(n\right)}\right)+F_{\left(n\right)}+\sum_{s=0}^{n-1}mK_{\left(s+\frac{1}{2}\right)}v_{\left(n-s-\frac{1}{2}\right)}\Delta t+R_{\left(n\right)}. \end{array}$$

Eq. **2** is propagated with the leapfrog algorithm:

[3] $$\begin{array}{c} \begin{array}{cc} v_{\left(n+\frac{1}{2}\right)} & =v_{\left(n-\frac{1}{2}\right)}+a_{\left(n\right)}\Delta t,\\ x_{\left(n+1\right)} & =x_{\left(n\right)}+v_{\left(n+\frac{1}{2}\right)}\Delta t. \end{array} \end{array}$$

This setup allows synchronization with MD data when Δt equals an integer multiple of δt, the integration time step of MD. More accurate multistep schemes for integrating stochastic dynamics exist (72, 73), but here, we adopt the simple leapfrog scheme because the autoregressive noise model of AIGLE benefits from a simple discretization scheme. Moreover, in consideration of the errors in the free energy calculations, the errors in autoregression, and the lack of a conservation law for stochastic dynamics, the numerical integration error is a minor issue as long as Δt is sufficiently small relative to the shortest vibrational period of the CVs.

The free energy G in Eq. **2** can be parameterized empirically, e.g., using a polynomial ansatz, or, more generally, it can be represented by a neural network ansatz when dealing with high-dimensional CVs. The noise term $\left\{R_{\left(n\right)}\right\}$ in Eq. **2** is modeled by a GAR model

[4] $$\begin{array}{c} R_{\left(n\right)}=\sum_{k=1}^{m_{A}}\phi_{\left(k\right)}R_{\left(n-k\right)}+\mu_{\left(n\right)}+\sigma_{\left(n\right)}w_{\left(n\right)}, \end{array}$$

where $\left\{\phi_{\left(k\right)}\right\}$ are Yule–Walker linear autoregressive parameters (74). $\mu_{\left(n\right)}$ and $\sigma_{\left(n\right)}$ are nonlinear functions that depend on the history of the noise. In our approach, $\mu_{\left(n\right)}$ and $\sigma_{\left(n\right)}$ are the outputs of a deep neural network whose arguments are $R_{\left(n-1\right)},\cdots ,R_{\left(n-m_{A}\right)}$. The residual noise $w_{\left(n\right)}$ represents the uncorrelated part of the noise on the scale of Δt, and should be close to Gaussian white noise for the scheme to be successful. The GAR model becomes a standard AR($m_{A}$) model (75) for constant $\mu_{\left(n\right)}$ and $\sigma_{\left(n\right)}$. When the time dependence of $\mu_{\left(n\right)}$ and $\sigma_{\left(n\right)}$ cannot be ignored, GAR outperforms AR in reducing $w_{\left(n\right)}$ to an almost ideal white noise upon training with MD data, a crucial property for the time correlation functions of CG dynamics to agree with the data.

Eqs. **2**–**4** constitute the AIGLE model. The corresponding AILE model is $ma_{\left(n\right)}=-\nabla G\left(x_{\left(n\right)}\right)+F_{\left(n\right)}+m\vartheta v_{\left(n-\frac{1}{2}\right)}+w_{\left(n\right)}$, where the friction satisfies $\vartheta =\sum_{s=0}^{n-1}K_{\left(s+\frac{1}{2}\right)}\Delta t$, and the white noise $w_{\left(n\right)}$ is fixed by the Markovian 2FDT. In AIGLE, the parameters defining G and F, the memory kernel, the Yule–Walker model, and the neural networks in the GAR model, are learned from MD trajectories kept near thermal equilibrium by a stochastic environment that mimics a heat bath. This is a common situation in realistic finite-temperature systems. Memory in these open systems should extend over a finite time interval, specified by the integer $m_{K}$, i.e., $K_{\left(s+\frac{1}{2}\right)}=0$ for $s>m_{K}-1$. $m_{K}$ and $m_{A}$ are *a priori* parameters of the same order of the relaxation time of the ACF of the velocities of the CVs. At the beginning of the learning protocol, it is convenient to set $m_{A}=m_{K}$ to be several times larger than the relaxation time. Upon fine tuning, the final values of $m_{A}$ and $m_{K}$ get close to the relaxation time of the velocity ACF, and we find empirically that a good choice corresponds to $m_{A}<m_{K}$. The mass m is fixed by the equipartition theorem.

The recommended learning procedure involves the three actions outlined below. More details are in the Methods section. In the first action, the models for G and the memory kernel $\left\{K_{\left(s+\frac{1}{2}\right)}\;|\;s\in \left[0,m_{K}-1\right]\right\}$ in Eq. **2** are trained on equilibrated MD data. Static and conservative forces $\left\{F_{\left(n\right)}\right\}$ are absorbed into G. Two steps are iterated to self-consistency. In the first step, G is optimized while keeping the memory kernel fixed. The loss function is the mean squared deviation from MD of the model prediction for the force on the CV without including noise effects. This procedure is equivalent to minimizing the noise. In the second step, the memory kernel is optimized, while keeping G fixed, by imposing orthogonality of velocity and noise in the least squared sense. Self-consistency typically requires a few thousand iterations. At the completion of the first action, the noise $\left\{R_{\left(n\right)}\right\}$ is defined by subtracting the gradient force, −∇G, and the memory-dependent friction force, predicted by the model, from the true force acting on the CV in the MD data. Then, we turn to the second action, in which the GAR model is optimized using a maximum likelihood loss function, in which the noise values $\left\{R_{\left(n\right)}\right\}$ constitute the time-series data. This procedure ends when the residual noise is almost white noise, and the GAR model is numerically stationary. At this point, the GLE is fully determined for equilibrium systems and can be used to model mesoscale dynamics under equilibrium conditions. However, when an external driving force F is present, extra training may be necessary. This is done in a third action, which is only executed if needed. In this procedure, the parameters that define G and F are refined with the loss function used in the first action, while keeping the memory kernel and the GAR model fixed. We tested the above procedure and the 1D AIGLE model on a toy system, the infinite harmonic chain, which can be solved analytically within the Mori–Zwanzig formalism. The results of this validation test, reported in *SI Appendix*, show that AIGLE reproduces with high accuracy the MD data, the analytical Mori–Zwanzig solution, and the 2FDT.

Finally, we generalize AIGLE for general lattice problems. First, we reinterpret Eq. **1** for on-lattice CVs. We let $x_{i}={\left(x_{i1},\cdots ,x_{id_{s}}\right)}^{T}$ represent a $d_{s}$-dimensional local order parameter associated to site-i of a $d_{l}$-dimensional Bravais lattice with periodic boundary conditions. L is the number of sites in the simulation supercell. By concatenating $\left\{x_{i}\right\}$ we define a $d_{s}L$-dimensional CV $x={\left(x_{1}^{T},\cdots ,x_{L}^{T}\right)}^{T}$. We let $A=\{A_{\mathrm{ij}}\}$ be the L×L adjacency matrix of the lattice. We use i∼j to indicate a neighboring pair ($A_{\mathrm{ij}}$=1). For large L, it is not practical to model a dense $d_{s}L\times d_{s}L$ memory kernel matrix $K\left(s\right)=\{K_{i\alpha }^{j\beta }\left(s\right)\}$ ($\alpha ,\beta \in [1,d_{s}]$) and a long-range correlated R(t) that preserve exactly the velocity correlation matrix or the 2FDT intrinsic to the data. The simplest approximation is to assume locality and set $K_{i\alpha }^{j\beta }\left(s\right)$ to be equal to zero whenever the indices i and j do not correspond to the same site, i.e., when i≠j. Even with this drastic approximation, the one-body memory kernel will depend not only on the autocorrelation but also on the cross-correlations of the CVs on different sites. Limiting consideration to close-neighbor correlations, we adopt a variational principle for the optimal memory kernel. We define the orthogonality tensor $\Omega_{i\alpha }^{j\beta }\left(t\right)=\left\langle R_{i\alpha }\left(t\right)v_{j\beta }\left(0\right)\right\rangle$, and define the corresponding orthogonality loss, a functional of K, by

[5] $$\begin{array}{c} L\left[K\right]\left(t_{K}\right)=\int_{0}^{t_{K}}\sum_{i,j,\alpha ,\beta }{\left|\Omega_{i\alpha }^{j\beta }\left(t\right)\right|}^{2}\left(\delta_{\mathrm{ij}}+A_{\mathrm{ij}}\right)dt. \end{array}$$

For a given cutoff of the memory time $t_{K}=m_{K}\Delta t$, the optimal one-body memory kernel $K^{*}$ minimizes Eq. **5**, i.e., $K^{*}={\mathrm{argmin}}_{K}L\left[K\right]\left(t_{K}\right)$. We call $K^{*}$ a “local kernel approximation” of the exact many-body memory kernel. It enforces a weak form of the 2FDT. The optimality condition for the memory kernel in the case of 1D AIGLE can be regarded as a special case of Eq. **5**. We show, in *SI Appendix*, that the local kernel approximation can be viewed as a special case of a general variational principle for the orthogonality condition. In practice, we still adopt the discretized form of Eq. **1** with the leapfrog scheme. We use a multidimensional version of Eqs. **2** and **3**, but keep the notation $f_{\left(n\right)}$ for a time-dependent function f(nΔt). We require $K_{\left(s+\frac{1}{2}\right)}=0$ for $s\geq m_{K}$. Given the force field F, after discretizing Eq. **5**, we obtain a least-square solution for the optimal memory kernel $K_{\left(s+\frac{1}{2}\right)}^{*}$ for $0\leq s<m_{K}$. The derivation, given in *SI Appendix*, is lengthy but straightforward. We also generalize the 1D GAR model to the multidimensional case. Note that, although $K^{*}$ is one-body, the noise R is still spatially correlated as required by the 2FDT. Thus, the noise generator can not be defined locally as commonly done with molecular dynamics thermostats. To deal with this complication, we allow the noise ${\left(R_{\left(n\right)}\right)}_{i}$ at site-i and time step n to depend not only on its own history but also on the history of a finite number of neighboring sites-j defined in terms of the adjacency matrix A. The sites-j could include nearest neighbor sites ($A_{\mathrm{ij}}=1$), next nearest neighbor sites, and so on. In other words, the multidimensional GAR model is analogous to a graph neural network (76) on the graph of the CVs defined by A. The details of the multidimensional GAR model are given in *SI Appendix*. The training of multidimensional AIGLE follows the same protocol of univariant AIGLE.

## 2. Applications

### A. Domain Wall as a Virtual Particle.

**Fig. 1.**
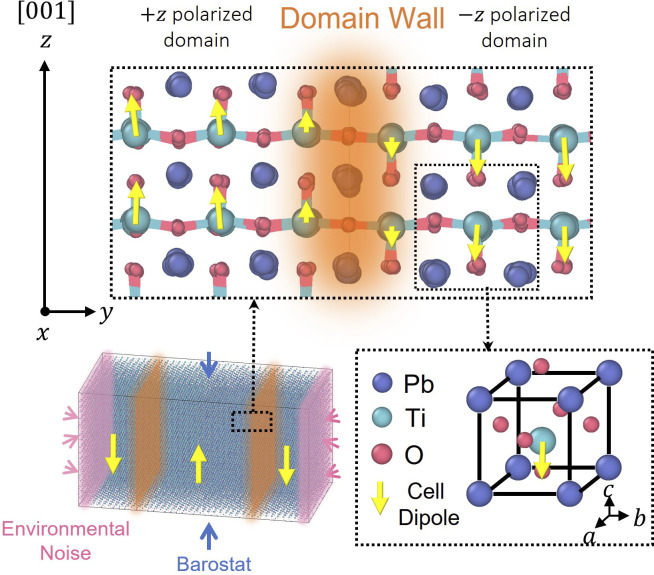
*Upper* panel: Lateral view of the 180° domain wall in PbTi${\text{O}}_{3}$. The bonds between titanium atoms and the nearest oxygen atoms are shown to visualize the domain separation. The yellow arrows represent the local dipoles, which are weaker near the domain wall. *Lower* panel (*Left*): A 20×40×20 supercell of PbTi${\text{O}}_{3}$ with parallel twin domain walls on the xz plane. The x and y dimensions are fixed to match the experimental lattice constant (see text) while the z dimension fluctuates under constant pressure $P_{z}=28$kbar (see text) at temperature T=300 K. *Lower* panel (*Right*): The elementary cell of PbTi${\text{O}}_{3}$.

In this section, AIGLE is used to study a prototypical problem of ferroelectric domain switching—the field-driven motion of a twin 180° domain wall in epitaxially grown PbTi${\text{O}}_{3}$. Schematic drawings of the domain wall and of the crystal structure are shown in Fig. 1. Electric dipole moments (local dipoles) $p_{j}$, represented by yellow arrows in Fig. 1, are associated with the Ti-centered elementary cells-j (77). The polarization is defined by $P=\sum_{j}p_{j}/V$, with V the volume of the sample. Polarization changes are experimentally observable. AIGLE is constructed from ab initio electronic structure data within DFT. Microscopic definitions of $p_{j}$ and P are given in *SI Appendix*.

In epitaxially strained tetragonal PbTi${\text{O}}_{3}$, polarized along the [001] crystallographic direction at room temperature (T=300 K), the ferroelectric domains have narrow 180° domain walls (77). A CV that describes continuously the switch of a domain from $+\hat{z}$ to $-\hat{z}$ is $\alpha =\sum_{i}\tanh\frac{p_{\mathrm{iz}}}{p_{*}}/2A_{\mathrm{wall}}$, where the sum extends to the local dipoles. We choose a value of $p_{*}$ that is close to the bulk average of $\|p_{\mathrm{iz}}\|$. In the simulations, we set the parameter $A_{\mathrm{wall}}$ to be equal to 400, the supercell area in the xz plane in units of elementary cells. With this definition, when the $+\hat{z}$ domain grows by one layer of unit cells in the y-direction, the increment of α is approximately equal to 1.

We model the motion of the domain wall driven by an external electric field $E=E\hat{z}$. Experimentally, the domain wall velocity, $v_{D}$, obeys approximately a phenomenological law suggested by Merz (58), according to which $v_{D}=v_{0}e^{-E_{a}/E}$, with $v_{0}$ and $E_{a}$ empirical parameters. For small E, $v_{D}\to 0$, and the wall dynamics is glassy. This motion, called domain wall creep, is usually initiated by the nucleation and growth of flat nuclei at the separating interface (78). On a coarser time scale, the moving interface can be viewed as a virtual particle that performs a succession of noise-activated, rare hopping events, rather than a steady continuous motion. This behavior cannot be deduced from phenomenological laws, like Merz’s, and is usually ignored in continuum models, but can be probed, in principle, with microscopic simulations (79). However, glassy dynamics can easily exceed all-atom simulation capabilities when the time scale is of the order of the microsecond or longer. To cope with the long-timescale bottleneck, one often turns to kinetic Monte Carlo (78), an approach that typically requires ad hoc iteration rules and assumes Markovianity. AIGLE can simulate non-Markovian dynamics with ab initio accuracy for time scales comparable to those reachable by kinetic Monte Carlo.

We generate training data for AIGLE with MD simulations of PbTi${\text{O}}_{3}$. We adopt the Deep Potential (DP) model for the interatomic interactions, and an effective Born charge (BC) model for the local dipoles (*SI Appendix*). The MD supercell is shown in Fig. 1, where the x and y dimensions are fixed to match the experimental lattice constant a=3.91 Å, and the z dimension is barostatted at a constant pressure of $P_{z}=28$ kbar, a value chosen to roughly match the experimental lattice constant c at 300 K and atmospheric pressure (*SI Appendix* and refs. 80 and 55 for more details). With the above setup, we run MD trajectories at T=300 K, with temperature controlled by a stochastic thermostat, in the presence of homogeneous electric fields of varying magnitude E, with 0≤E≤3mV/Å, along the $\hat{z}$ direction. The microscopic data for the CV α are extracted from these trajectories. In these simulations, the atomistic degrees of freedom equilibrate quickly with the environment, and the loss of detailed balance is mostly associated with the CV describing domain motion.

The atomistic simulations suggest that the dynamics of α resemble that of a virtual particle subject to colored noise in a tilted periodic potential (81). When E is small, the particle is trapped in a metastable equilibrium and the velocity ACF of α, defined by $A_{\dot{\alpha }\dot{\alpha }}\left(\tau \right)=\left\langle \dot{\alpha }\left(\tau \right)\dot{\alpha }\left(0\right)\right\rangle$, exhibits several characteristic oscillations (modes), i.e., a behavior dramatically different from the simple exponential decay characteristic of Brownian dynamics driven by white noise. These characteristic modes originate mainly from the optical phonons of PbTi${\text{O}}_{3}$ and provide the thermal fluctuations that activate nucleation-driven creep events at small driving fields. Taking time scale separation into account, it is convenient, when constructing a CG model, to filter out the frequencies much higher than that of the slowest mode of α (≈30 $cm^{-1}$). Then, a new CV, x, is constructed by acting on α with a truncated Gaussian filter in time:

[6] $$\begin{array}{c} x_{\left(n\right)}=\sum_{q=0}^{3\varsigma }\frac{\exp(-\frac{q^{2}}{2\varsigma^{2}})}{\sum_{q=0}^{3\varsigma }\exp\left(-\frac{q^{2}}{2\varsigma^{2}}\right)}\alpha \left(t=n\Delta t-q\delta t\right). \end{array}$$

Here, ς is the truncation parameter that we set equal to 40. With this choice, the modes of α in the range $\left[0,100\right]\;cm^{-1}$ are barely affected, while the modes with higher frequency are suppressed (*SI Appendix*). The AIGLE integration time step Δt=10 fs is equal to five times the MD time step δt=2 fs. This procedure is substantiated by the fact that the residual noise, after training the model, is indeed very close to white noise. The GLE of motion for x, deriving from Eq. **2**, is

[7] $$\begin{array}{c} a_{\left(n\right)}=-\partial_{x}U\left(x_{\left(n\right)}\right)+pE+\sum_{s=0}^{n-1}K_{\left(s+\frac{1}{2}\right)}v_{\left(n-s-\frac{1}{2}\right)}\Delta t+\frac{1}{m}R_{\left(n\right)}. \end{array}$$

Here, we parameterized the FES of Eq. **2** with the periodic function $G\left(x\right)=mU\left(x\right)=mU_{b}\tanh\left(k\left(1-\cos\omega \left(x-x_{0}\right)\right)\right)$. $m\approx 1.0\times 10^{3}$ amu is a scalar mass, estimated with the equipartition theorem. $U_{b}$, the barrier height, is predetermined with metadynamics (62) since it is hard to fit it accurately in the near-equilibrium regime without enhanced sampling. The external force in Eq. **2** is represented by F(x)=mpE. The values of the parameters k, ω, $x_{0}$, and p, are fixed by training. The time cutoffs for the memory kernel K and for the GAR model for R are $m_{K}\Delta t=2$ ps and $m_{A}\Delta t=0.4$ ps, respectively. Assuming a linear response regime, the model parameters are independent of E. The AIGLE model introduced here is trained on several MD trajectories with E∈[2.0,2.4] mV/Å. Details of training and validation can be found in *SI Appendix*. MD systems are at metastable equilibrium for E≈2mV/Å and near equilibrium for smaller E.

**Fig. 2.**
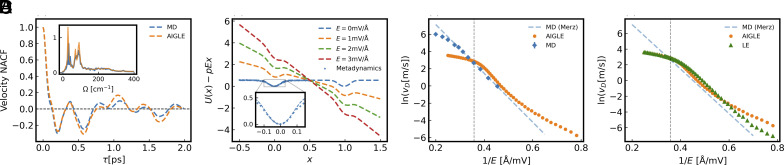
(*A*) The velocity NACF $C_{\mathrm{vv}}^{\text{MD}}\left(\tau \right)$ from MD (blue) and the velocity NACF $C_{\mathrm{vv}}^{\text{GLE}}\left(\tau \right)$ from AIGLE (orange), for τ<2ps. For τ>2ps the correlations are confined to the interval [−0.1,0.1] and decay rapidly to zero. The *Inset* reports $R({\hat{C}}_{\mathrm{vv}}^{\text{MD}}\left(\Omega \right))$ (blue) and $R({\hat{C}}_{\mathrm{vv}}^{\text{GLE}}\left(\Omega \right))$ (orange) in arbitrary units. The first peak of $R({\hat{C}}_{\mathrm{vv}}^{\text{GLE}}\left(\Omega \right))$ is located near 30 $cm^{-1}$. (*B*) Free energy profiles along the CV x in the presence of driving fields of various strengths. Two periods of the parametrized U(x) (see text) are shown. U(x) is represented by a dashed blue line. Metadynamics results are also reported as blue crosses, for comparison. The *Inset* shows a magnified plot of the free energy basin including U(x) and metadynamics data. (*C*) The natural logarithm of the domain wall velocity $v_{D}$ is plotted against 1/E. The vertical dashed line indicates $E=E^{*}$. Each GLE data point (orange dot) is the average of five AIGLE simulations lasting 0.5 μs each. The corresponding error bars are smaller than the size of the dot. Each MD data point (blue diamond) is the average of 100 MD trajectories lasting 0.1 ns each. The error bars are smaller than the size of the diamond. The dashed blue line is the best fit of Merz’s law with MD data ($E_{a}=28\;\mathrm{mV}/$Å). (*D*) A comparison of AIGLE and LE predictions for $\ln v_{D}$ vs. 1/E. Each LE data point (green triangle) is the average of five LE trajectories lasting 0.5 μs each. The error bars are smaller than the size of the triangle.

Comparison of $C_{\mathrm{vv}}^{\text{MD}}\left(\tau \right)$, the normalized autocorrelation function (NACF) of the CV velocity extracted from MD, with its AIGLE counterpart, $C_{\mathrm{vv}}^{\text{GLE}}\left(\tau \right)$, provides a direct validation of AIGLE. The two NACFs, calculated at metastable equilibrium conditions for E=2 mV/Å, are reported in Fig. 2*A*. They agree well with each other but for minor discrepancies. The figure also indicates that the adopted cutoff $m_{K}$ is large enough to satisfy the condition $C_{\mathrm{vv}}^{\text{MD}}\left(\tau >m_{K}\Delta t\right)\ll 1$. A major cause of the small differences between MD and AIGLE is apparent in the inset of Fig. 2*A*, which reports the real parts of Fourier transforms of the velocity NACFs, $R({\hat{C}}_{\mathrm{vv}}^{\text{MD}}\left(\Omega \right))$ and $R({\hat{C}}_{\mathrm{vv}}^{\text{GLE}}\left(\Omega \right))$. The slowest mode occurs at Ω≈ 30 $cm^{-1}$ in both NACFs. In $R({\hat{C}}_{\mathrm{vv}}^{\text{MD}}\left(\Omega \right))$ this mode exhibits a fast oscillatory line shape, indicating relaxational origin. The same mode in $R({\hat{C}}_{\mathrm{vv}}^{\text{GLE}}\left(\Omega \right))$ has a smooth Lorentzian line shape with a peak frequency that matches the harmonic frequency of the nearly quadratic free energy basin depicted in Fig. 2*B*. This indicates that the relaxational fluctuation of the domain wall is turned into an effective harmonic oscillation in a potential well. Since the ansatz for U(x) assumes a smooth, rather than fractal, dependence on x, the slowest mode of $R({\hat{C}}_{\mathrm{vv}}^{\text{GLE}}\left(\Omega \right))$ displays a clean harmonic peak, sharper than the relaxational peak of $R({\hat{C}}_{\mathrm{vv}}^{\text{MD}}\left(\Omega \right))$. This subtle difference is, in fact, a desired consequence of coarse-graining the FES. Two other modes (near 80 $cm^{-1}$) are displayed by $R({\hat{C}}_{\mathrm{vv}}^{\text{GLE}}\left(\Omega \right))$ and by $R({\hat{C}}_{\mathrm{vv}}^{\text{MD}}\left(\Omega \right))$ as well. At higher frequencies, the spectrum of $R({\hat{C}}_{\mathrm{vv}}^{\text{GLE}}\left(\Omega \right))$ is quite smooth and agrees well with the MD results.

The optimized free energy profile as a function of x is shown in Fig. 2*B*. Metastability disappears for E greater than $E^{*}\approx 2.8$ mV/Å when the profile becomes monotonic. Hence, $E^{*}$ represents the threshold beyond which the near-equilibrium regime appropriate for AIGLE is no longer valid. Under near-equilibrium conditions, the lifetime of a metastable state should be much longer than the relaxation times of the atomic vibrations. To study this phenomenology, we run AIGLE for a dense grid of electric field values in the interval (1,5) mV/Å. To visualize the variation of the domain velocity $v_{D}$, which spans several orders of magnitude, we display in Fig. 2*C* the natural logarithm of $v_{D}$, extracted from AIGLE and MD simulations, respectively, as a function of 1/E. MD data are only available for $v_{D}⪆1$m/s due to time limits of fully atomistic simulations. When AIGLE and MD data are both available, the two approaches agree well for $E\leq E^{*}$, i.e., under near-equilibrium conditions. For $E>E^{*}$, the domain velocity of MD is significantly larger than its AIGLE counterpart. From a coarse-graining point of view, this occurs because the 2FDT, valid near equilibrium, has been imposed far from equilibrium. From a microscopic point of view, the electric dipoles, temporarily associated with the moving domain wall, are unable to dissipate energy before separating from the wall. Far from equilibrium memory effects are different from those learned for $E<E^{*}$. Thus, the present AIGLE model should only be used when $E\leq E^{*}$, i.e., within the creep regime of the Markovian theory of elastic interface dynamics (59, 60). When $v_{D}⪆1$m/s, MD shows linear behavior of $\ln v_{D}$ with 1/E, in agreement with Merz’s law: $\ln v_{D}=\ln v_{0}-E_{a}/E$ (58). A best fit of the MD data to this law gives $E_{a}=28\;\mathrm{mV}/$Å. AIGLE gives essentially the same result, $E_{a}=27\;\mathrm{mV}/$Å, for E∈[2mV/Å, $E^{*}]$. Thus, $v_{D}$ at low electric fields can be estimated from Merz’s law fitted to MD for $v_{D}⪆1$ m/s, as done, e.g., in refs. 78 and 79. However, when $v_{D}\ll 1$m/s, direct AIGLE simulations display a gradual deviation from Merz’s law, as illustrated in Fig. 2*C*. When 1/E>0.6 Å/mV, AIGLE predicts a $v_{D}$ higher than Merz’s law by orders of magnitude. This behavior is similar to the stretched exponential inferred from the relation $\ln\left(v_{D}/v_{0}\right)\propto {\left(E/E_{c}\right)}^{-\mu }$ of the Markovian theory of elastic interfaces (59) when the dynamic exponent μ is less than 1. This theory assumes a Markovian overdamped regime. Yet, the deviation from Merz’s law, predicted by AIGLE at low fields, is markedly more rapid than the stretched exponential of the Markovian theory. This suggests that memory and inertia play an increasingly important role in the regime of very rare domain motions.

To gauge the implication of non-Markovianity, we approximate AIGLE with AILE. Fig. 2*D*, shows that LE predicts for $v_{D}$ a behavior consistent with Merz’s law, which is not surprising because the derivation of Merz’s law requires a Markovian approximation. The same figure shows that LE and AIGLE agree well with each other when 1/E is close to $1/E^{*}$, a situation in which the external driving force dominates over memory and noise. For larger 1/E, the LE predicted behavior deviates from a pure exponential in a very minor way, underestimating $v_{D}$ by orders of magnitude relative to AIGLE at the largest values of 1/E. Within Markovian dynamics, the friction is always dissipative, hindering thermally activated motion irrespective of the time scale of the creep events. An even simpler dynamics is postulated in the Markovian theory of elastic interfaces (59, 60) that adopts an overdamped Langevin equation, where both memory and inertia effects are absent. By contrast, within AIGLE, memory results from a convolution of oscillating functions and can occasionally lead to a kinetic energy increase over a short time interval. In combination with inertia, this effect enhances the likelihood of barrier crossing. From the perspective of transition state theory, this effect can be understood as effectively enhancing the pre-exponential factor in the formula for the rate. Non-Markovian effects that facilitate barrier-crossing have also been discussed in other contexts, such as, e.g., in the Grote-Hynes theory of chemical reaction rates (82).

Using the domain velocity $v_{D}\left(E\right)$ calculated with AIGLE, we can estimate the hysteresis loop observed experimentally when the polarization is reversed by a driving field. We report in *SI Appendix* a hysteresis loop calculation using a very simple model of ferroelectric switching that ignores point defects and dependence on the curvature of the domain wall. The results are in semiquantitative agreement with experiments.

### B. CG Lattice Dynamics.

Here, we use multidimensional AIGLE to describe the dynamics of lattice CVs, which are either the local dipole moments $\left\{p_{j}\right\}$ or a CG model of them. The underlying microscopic model is the all-atom DP model of Section A. For each atomic configuration, the local dipoles are provided by a neural network model (*SI Appendix*).

**Fig. 3.**
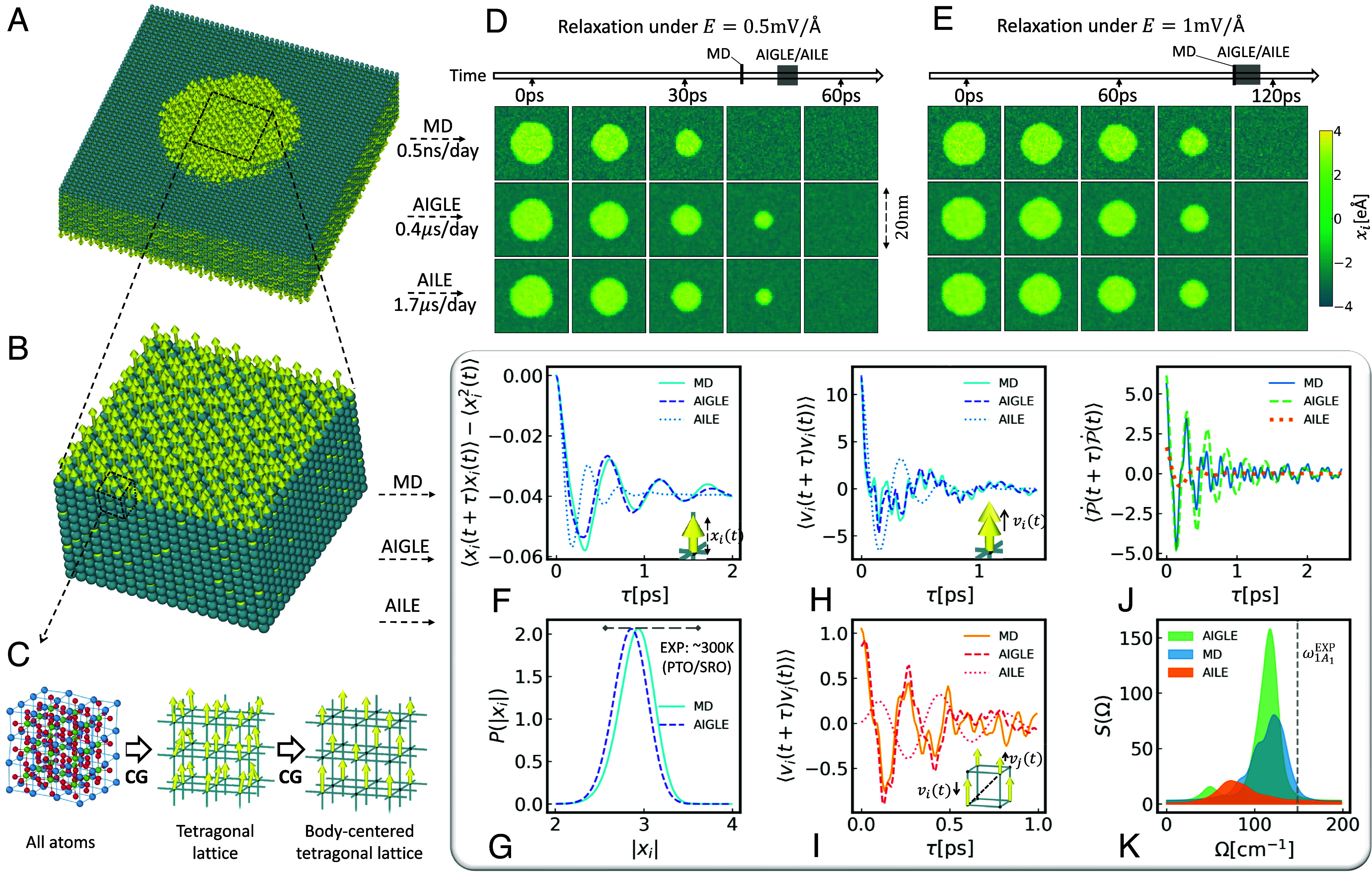
(*A*) A (nonequilibrium) configuration of PbTi${\text{O}}_{3}$ showing a cylindrical up-polarized domain in an environment of opposite polarization. Spheres are Ti atoms, Pb and O atoms are not shown. The arrows depict the local dipole moments assigned to Ti-centered elementary cells. (*B*) A configuration within the up-polarized domain. (*C*) Coarse-graining procedure. (*D* and *E*) Relaxation dynamics of the local dipoles in an atomic layer perpendicular to the direction $\hat{z}$ of spontaneous polarization under electric fields E=0.5 mV/Å (*D*), and E=1 mV/Å (*E*). The three horizontal sequences of panels in (*D*) and (*E*) depict the evolution of the yellow domain from MD, AIGLE, and AILE. The pixels correspond to the S sites. In the MD panels, they give the magnitude and sign of the microscopic dipoles $\left\{p_{\mathrm{jz}}\right\}$, as per scale on the right. In the AIGLE and AILE panels the pixels associated with the $S_{1}$ sites give the CG dipoles $\left\{x_{j}\right\}$, while those associated with the $S_{2}$ sites are the average of the CG dipoles at the neighboring $S_{1}$ sites. The lifetimes of the cylindrical domain are shown on the two time axes. The solid vertical bars, at 40 ps for E=0.5 mV/A, and at 104 ps for E=1 mV/A, are extracted from two MD simulations. The gray rectangles are extracted from nine independent AIGLE and AILE simulations for each value of the electric field. (*F*) Shifted autocorrelation function (ACF) of the local dipole $x_{i}\left(t\right)$, in units of (eÅ)^2^, from MD, AIGLE, and AILE. (*G*) Equilibrium probability distribution $P(|x_{i}|)$ of the dipole magnitude from MD and AIGLE. The AILE result coincides with AIGLE and is not reported. The gray dashed line shows the range of the average dipole magnitude $\left\langle |x_{i}\left(t\right)|\right\rangle$ from different experiments (83–85). (*H*) ACF of the time derivative, $v_{i}\left(t\right)$, of $x_{i}\left(t\right)$, in units of (eÅ/ps)^2^. (*I*) Cross-correlation function of $v_{i}\left(t\right)$ and $v_{j}\left(t\right)$ on adjacent sites in the $S_{1}$ lattice, in units of (eÅ/ps)^2^. (*J*) ACF of $\dot{P}\left(t\right)$, the time derivative of the polarization P(t), in units of ${\left(\mu C/cm^{2}/ps\right)}^{2}$. (*K*) Gaussian convoluted Fourier transform S(Ω) of the ACF of $\dot{P}\left(t\right)$ (see text for details). The gray dashed line indicates the peak frequency $\omega_{1A_{1}}^{\mathrm{EXP}}$ of the Raman spectroscopy feature associated to the zone-center $1A_{1}$ transverse optical phonon (86).

We run NVT-MD to generate the training data. The lattice parameters are fixed to a=b=3.93 Å and c≈4.04 Å. For E=0, we run equilibrium NVT-MD in a 8nm×8nm×5nm supercell comprising a single ferroelectric domain. The system is illustrated in Fig. 3*B*, where yellow arrows represent the local dipole moments. For E=0.5 mV/A and E=1 mV/A along +z, we run near equilibrium NVT-MD in a 20nm×20nm×5nm supercell that initially contains two opposite ferroelectric domains, i.e., a nearly cylindrical up(+z)-polarized domain having a radius of about 6nm, embedded in an environment of opposite polarization, as illustrated in Fig. 3*A*. To reduce the energy cost of the cylindrical interface the up-polarized domain shrinks during the simulation, in spite of the applied field favoring up-polarization, with a longer relaxation time when E is larger. In the MD simulations, we calculate the trajectories of all the local dipoles $\left\{p_{j}(t)\right\}$. Taking the local dipoles in the tetragonal PbTi${\text{O}}_{3}$ lattice as CVs (Fig. 3*C*, *Middle*), the degrees of freedom are one-fifth of the atom coordinates (Fig. 3*C*, *Left*). Further coarse-graining is motivated by the following considerations. The time correlations of the Cartesian components of the local dipole velocities, i.e., $\left\langle {\dot{p}}_{j\alpha }\left(t_{0}+\tau \right){\dot{p}}_{j\beta }\left(t_{0}\right)\right\rangle$ for α,β∈{x,y,z}, indicate that the correlations for α≠β are negligible compared to those for α=β. Thus, we can reduce by one-third the CVs by retaining only the z-components, $\left\{p_{\mathrm{jz}}\left(t\right)\right\}$, of the local dipoles, which are related to spontaneous polarization. Nearest neighbor dipoles are strongly correlated, because the oxygen atoms, whose displacements contribute to the polarization the most, are shared between adjacent cells. As a consequence, further coarse-graining is possible by blocking into a single dipole pairs of nearest-neighbor dipoles of the original simple tetragonal lattice S. The blocking operation defines two interpenetrating body-centered tetragonal (BCT) lattices $S_{1}$ and $S_{2}$ obtained from S by bipartition. We assume that our choice of CG dipoles corresponds to $S_{1}$, as illustrated in the right panel of Fig. 3*C*. If $a=a\hat{x}$, $b=b\hat{y}$, $c=c\hat{z}$ are the (conventional) unit cell vectors of S, the (conventional) unit cell vectors of $S_{1}$ are u=a+b, v=−a+b, and w=2c. Let L and A be the size and the adjacency matrix, respectively, of $S_{1}$. Each site-i of $S_{1}$ has 12 neighboring sites (in the sense of graph adjacency on $S_{1}$), displaced by (±a±b), (±a±c), and (±b±c), respectively. The corresponding CVs are denoted by $\tilde{p}={\left(p_{\mathrm{iz}}\right)}_{i\in [1,L]}$. By construction, the degrees of freedom in $\tilde{p}$ are one-thirtieth of the atomic coordinates, but the polarization along $\hat{z}$ is left unaffected. Then, we apply a truncated Gaussian filter in time to $\tilde{p}\left(t\right)$ to remove high-frequency contributions. The resulting CVs are called $\left\{x_{\left(n\right)}\right\}$:

[8] $$\begin{array}{c} x_{\left(n\right)}=\sum_{l=0}^{3\varsigma }\frac{\exp(-\frac{q^{2}}{2\varsigma^{2}})}{\sum_{l=0}^{3\varsigma }\exp\left(-\frac{q^{2}}{2\varsigma^{2}}\right)}\tilde{p}\left(t=n\Delta t-q\delta t\right). \end{array}$$

Here δt=2fs, Δt=5δt and ς=40, as in Section A. The polarization of the system is $P\left(t=n\Delta t\right)=2\sum_{i}x_{i,(n)}/V$.

The AIGLE model for $\left\{x_{\left(n\right)}\right\}$ is

[9] $$\begin{array}{c} \begin{array}{cc} Ma_{\left(n\right)} & =-\nabla_{x}G\left(x_{\left(n\right)}\right)+pME_{\left(n\right)}\\ & \;+\sum_{l=0}^{n-1}MK_{\left(l+\frac{1}{2}\right)}v_{\left(n-l-\frac{1}{2}\right)}\Delta t+R_{\left(n\right)}, \end{array} \end{array}$$

under local kernel approximation. We use for multidimensional AIGLE the same notation adopted in Eq. **7** for one-dimensional AIGLE. In principle, the external field $E_{\left(n\right)}$ can vary in space and time, but we consider here only fields that are time-independent and uniform in space. For the free energy G(x) we assume a simple polynomial form, $G\left(x\right)=\sum_{i}\left(b_{1}x_{i}^{2}+b_{2}x_{i}^{4}\right)+\sum_{\mathrm{ij}}A_{\mathrm{ij}}b_{\mathrm{ij}}{\left(x_{i}-x_{j}\right)}^{2}$, suggested by effective Hamiltonian models (55). By symmetry, $b_{\mathrm{ij}}=b_{3}$ if the sites i and j are separated by (±a±b), and we set $b_{\mathrm{ij}}=b_{4}$, otherwise. The b coefficients are assumed to be independent of E, as appropriate in the linear response regime. Hence, we limit simulations to E≤1 mV/A. Our model for G is short-ranged but captures well the dipole–dipole interactions of the DP model within the cutoff radius of the latter. Long-range electrostatic interactions among the dipoles have a negligible effect on the ferroelectric transition in PbTi${\text{O}}_{3}$ (see, e.g., ref. 80 and references therein).

Training proceeds through several steps. We first predetermine G with equilibrium MD data (E=0) by force matching. Then, we calculate the memory kernel with the same data under local kernel approximation and train the multidimensional GAR model using $R_{\left(n\right)}$ as time series data. For the noise at site-j, the GAR model includes the noise history of site-j and of its neighbors on $S_{1}$ displaced by (±a±c), (±b±c) or (±2c). In the last step, we retrain G and p with nonequilibrium MD data (E>0). The details are in *SI Appendix*. The corresponding AILE model is defined by the Markovian approximation of AIGLE as in the one-dimensional case.

The relaxation dynamics of the cylindrical domain in Fig. 3*A*, under weak applied field, is illustrated in Fig. 3*D*, for E=0.5 mV/A, and in Fig. 3*E*, for E=1 mV/A. The noise in AIGLE and AILE trajectories is at the origin of the observed fluctuations in the domain lifetime. Within the uncertainty of the noise, AIGLE and AILE lifetimes coincide, suggesting that non-Markovian effects should be negligible. Indeed, domain shrinking is caused primarily by surface tension, which acts to reduce the area of the interface between domains, a systematic effect originating from the gradient of the free energy. The MD lifetime is deterministic and is extracted from a single trajectory. It agrees with AIGLE/AILE within the uncertainty of the noise for E=1 mV/A (Fig. 3*E*), but is approximately 10ps shorter than AIGLE/AILE for E=0.5 mV/A (Fig. 3*D*). This discrepancy is likely due to the inaccuracy of the simple polynomial model adopted for the FES. Non-Markovian effects should be more pronounced for larger cylindrical domains, where the surface tension is smaller. Simulation of much larger domains would be feasible with AIGLE and AILE but not with all-atom MD, hampering direct comparison for these settings. The special case of a planar domain wall dynamics under applied field was considered in Section A, where it was found that non-Markovian effects play a role for very weak fields.

Next, we consider a uniformly polarized bulk sample in the absence of an external field (E=0). Static and dynamic properties of the dipoles are reported in Fig. 3*F*–*K*. Memory and noise effects are more pronounced in the equilibrium dynamics of the bulk than in the relaxation dynamics of a cylindrical interface. Indeed, the MD ACF of an individual CG dipole $x_{i}$ is reproduced accurately by AIGLE but not by AILE (Fig. 3*F*). At the same time, nearly identical results are obtained with AIGLE and AILE for static properties like the probability distribution $P(|x_{i}|)$ of the local dipole, reported in Fig. 3*G*, as expected from the fact that AIGLE and AILE yield the same equilibrium Boltzmann distribution. On the scale of Fig. 3*G*, AIGLE and AILE are identical and only AIGLE is reported. The AIGLE distribution overlaps almost perfectly with the MD distribution barring a minor overall shift, much smaller than the range of the average dipole magnitudes extracted from experiments.

The remaining panels in the figure confirm the importance of non-Markovian effects. Fig. 3*H* shows that the ACF of $v_{i}\left(t\right)$, the time derivative of $x_{i}\left(t\right)$, is reproduced accurately by AIGLE but not by AILE. Also, the cross-correlation function between the time derivatives of neighboring dipoles shown in Fig. 3*I* is reproduced well, at least up to about 0.5 ps, by AIGLE but not by AILE. These results suggest that the adopted local kernel approximation, which uses an optimized one-body memory kernel and many-body-correlated noise, can capture the short-range correlations among the dipoles that should dominate the fluctuation and dissipation of observables like the spontaneous polarization P. Indeed, the ACF of $\dot{P}\left(t\right)$, the time derivative of P(t), displayed in Fig. 3*J*, shows that AIGLE captures its dominant oscillatory frequency, while AILE misses it completely. However, at larger lagging times τ in the interval [0.5,1.5]ps AIGLE fails to reproduce the weak out-of-phase oscillations observed in MD. This behavior may originate from anharmonic couplings between vibrational modes that are not captured in the CG model. Neglect of long-range correlations in the noise could be another source of errors, as suggested by the observation that AIGLE would overestimate $\left\langle {\dot{P}}^{2}\left(t\right)\right\rangle$ by about 30% if the GAR model did not include the history dependence of neighbors separated by (±2c). Thus, including longer-range correlations may improve the accuracy of the model. This may be possible by adopting a more elaborate GAR model for the noise while retaining the simple local kernel approximation of AIGLE. It is also instructive to compute S(Ω), the spectrum of $\left\langle \dot{P}\left(t+\tau \right)\dot{P}\left(t\right)\right\rangle$, which can be compared with experimental infrared spectroscopy. The spectra from MD, AIGLE, and AILE, given by $S\left(\Omega \right)=R\int_{0}^{\infty }d\tau \exp\left(-i\Omega \tau \right)\left\langle \dot{P}\left(t+\tau \right)\dot{P}\left(t\right)\right\rangle$, are reported in Fig. 3*K*, upon Gaussian broadening with full width at half maximum of $12\;{\mathrm{cm}}^{-1}$. As expected from the real-time data, the AILE peak in Fig. 3*K* is significantly weaker than the other two, while AIGLE is stronger than MD, reflecting a sharper spectral feature. AIGLE reproduces well the peak frequency of MD, while AILE is red-shifted by approximately $40\;{\mathrm{cm}}^{-1}$. The spectral feature in Fig. 3*K* is associated with the zone-center $1A_{1}$ transverse optical phonon, which is both infrared and Raman active. The corresponding feature from Raman scattering experiments lies at $\omega_{1A_{1}}^{\mathrm{EXP}}=148.5\;{\mathrm{cm}}^{-1}$ (86), with a full width at half maximum (FWHM) of approximately $30\;{\mathrm{cm}}^{-1}$, while the MD FWHM is $43\;{\mathrm{cm}}^{-1}$ and that of AIGLE is $23\;{\mathrm{cm}}^{-1}$. The red shift of the MD/AIGLE peak at $120\;{\mathrm{cm}}^{-1}$, relative to the experiment, is mainly due to the adopted DFT approximation.

The above results show that AIGLE with the local kernel approximation can capture to a large extent the dynamic behavior of the CVs predicted by MD for bulk PbTi${\text{O}}_{3}$. At the same time, AILE, while equivalent to AIGLE for static properties, can not capture dynamical correlations when memory is important.

Finally, a comment on computational efficiency is in order. When modeling the dynamics of a 20nm×20nm×5nm supercell on one Nvidia-A100 GPU, MD runs at 0.5 ns/d, AIGLE at 0.4μs/d, and AILE at 1.7μs/d. Thus, the speedup over MD is of three orders of magnitude for both AIGLE and AILE. Moreover, AIGLE and AILE use significantly less memory than MD, facilitating simulations of significantly larger supercells.

## 3. Discussion

We have introduced a practical scheme to construct CG GLE models from MD trajectories. Our approach does not rely on the formal projection of MD onto the space of the CVs. As a consequence, the GLE construct is not exact, but should rather be viewed as a physically motivated approximation. While the idea of parameterizing GLE models with data extracted from MD trajectories dates back to at least 50 y (28), we exploit here modern techniques, such as machine learning and deep neural network representations, to generate extensive training datasets with MD and to construct the correlated noise model in the GLE. This enables us to construct AIGLE models, consistent with the microscopic dynamics, for one-dimensional and multidimensional CVs. Multidimensional AIGLE is not a trivial extension of its one-dimensional counterpart, and requires a local variational approximation for the memory kernel and a nearsightedness approximation for the correlated noise. The latter could be formulated only for systems in which local CVs reside on sites with a fixed topology described by an adjacency matrix or a graph, such as crystals and individual polymeric molecules. How to extend the approach to more general disordered systems remains an open issue. Here, we considered mesoscale processes in PbTi${\text{O}}_{3}$, a ferroelectric crystal, to illustrate the scheme and test its validity.

When used to study one-dimensional interface dynamics, AIGLE can model rare events on glassy landscapes caused by nucleation and growth at the atomistic level, reproducing the interface evolution driven by a weak applied field at a much lower computational cost than MD. In contrast to MD, AIGLE can access very rare events, revealing that, in the “slow” creep regime, when the time scale of the events is much longer than that of the memory, the scaling law for the domain velocity may deviate significantly from that of the “fast” creep regime, due to non-Markovian effects.

When applied to the dynamics of extensive CVs, AIGLE can model the relaxation of an elastic interface of any shape, a special case of extended defects, while still keeping the bulk dynamics of the CVs consistent with MD. These features distinguish AIGLE from other multiscale models with more drastic levels of coarse-graining, such as, e.g., a Landau–Ginzburg field theory of the extensive CVs in the continuum limit. A field theory model can not provide atomistic level resolution of an interface, or correctly describe the vibrational spectrum of a global order parameter like the electric polarization at low but nonzero frequency. In ferroelectric materials polarization dynamics at low frequency is typically dominated by an optical phonon mode that cannot be reduced to white noise, and cannot be modeled by AILE. In this context, AIGLE captures many-body correlations between CV components that are topologically close when the distance is measured in a graph. This feature is the key difference between a truly multidimensional GLE and a set of one-dimensional GLEs with independent frictions and noises.

Our study provides also examples where non-Markovian effects are irrelevant. In the CG lattice dynamics of PbTi${\text{O}}_{3}$, AIGLE and AILE give similar results for the motion of an interface dominated by a systematic driving force like the surface tension. In that case, memory effects are negligible, but our study shows that they may become important for glassy dynamics. It would be interesting to investigate the effect of driving fields that vary in space and time. Terahertz control of materials is an area of growing importance due to novel experimental developments (87). For controlling fields within the frequency range of atomic/molecular vibrations, non-Markovian memory and noise effects could be in resonance with the external controlling field, coupling the latter to collective behavior associated with domain motion and/or phase transitions.

Modeling CG lattice dynamics with AIGLE or AILE brings us to a scale where phenomena are typically treated with continuum models. These phenomena include general domain dynamics and phase separation in the condensed phase, which occur in ferromagnets (88), ferroelectrics (89), and alloys (9, 10). Other phenomena, important in the fabrication and characterization of nanomaterials, include morphology evolution in epitaxial growth (90) and height fluctuations of two-dimensional membranes (91). In these contexts, the application of phase field models is very popular, whereby a continuum approximation is imposed a priori and partial differential equations are constructed, guided by symmetry and physical intuition. This approach often captures the correct qualitative physics. However, when defects like impurities, grain boundaries, and domain walls are present, ad hoc continuum approximations fitted to few experimental observations, may be insufficient. When the role of defects is important, lattice models for the local dipole moments, local strains, and spins should be more reliable, as defect dynamics could be incorporated in lattice models by coupling homogeneous CVs on a lattice to a finite number of virtual particles representing mobile defects. Along this line, one may be able to model notoriously difficult processes, such as those leading to the fatigue of ferroelectric devices when the dynamics of point defects gradually impacts the dynamics of domain walls over large space and time scales (92).

All the applications discussed in the present work focused on the near-equilibrium regime, where the dynamics is constrained by the 2FDT. However, our methodology could be extended to far-from-equilibrium situations, where a governing principle like the 2FDT does not apply. A regression-based approach like AIGLE can be adapted to deal with these situations, whereas conventional approaches based on ACFs would lose the convenience of direct construction of memory and noise terms. How to extend AIGLE to deal with far-from-equilibrium phenomena is a direction that we intend to explore in future studies.

## 4. Materials and Methods

Here, we illustrate the learning procedure for the univariant GLE (Eqs. **2**–**4**). We will use $F_{\left(n\right)}$ as an abbreviation for $-\nabla G\left(x_{\left(n\right)}\right)+F_{\left(n\right)}$. Also, without loss of generality, we assume m=1.

### Separation of Noise.

The first step of learning relies on equilibrated MD trajectories $\kappa =\{x_{\left(n\right)}|n\in \left[0,N\right]\}$ with ergodic fast degrees of freedom. $v_{\left(n+\frac{1}{2}\right)}$ and $a_{\left(n\right)}$ are computed from Eq. **3**. $v_{\left(n\right)}$ is further determined as the average of $v_{\left(n+\frac{1}{2}\right)}$ and $v_{\left(n-\frac{1}{2}\right)}$. We turn the ensemble-averaged orthogonality condition $\langle R_{\left(n\right)}v_{\left(0\right)}\rangle =0$ to a time-averaged one. To achieve that, we introduce the shifted GLE with an arbitrary starting point $n_{0}\geq 0$:

[10] $$\begin{array}{c} a_{\left(n\right)}=F_{\left(n\right)}+\sum_{s=0}^{n-n_{0}-1}K_{\left(s+\frac{1}{2}\right)}v_{\left(n-\frac{1}{2}-s\right)}\Delta t+{\tilde{R}}_{\left(n\right)}^{\left(n_{0}\right)}. \end{array}$$

Here, ${\tilde{R}}_{\left(n\right)}^{\left(n_{0}\right)}$ is a shifted noise and $n>n_{0}$ is required. As demonstrated in Ref. (27), $\left\langle {\tilde{R}}_{\left(n_{0}+k\right)}^{\left(n_{0}\right)}{\tilde{R}}_{\left(n_{0}\right)}^{\left(n_{0}\right)}\right\rangle =\left\langle R_{\left(n+k\right)}R_{\left(n\right)}\right\rangle$ for a stationary noise series when n→∞.

For a given CV trajectory, the shifted noise ${\tilde{R}}_{\left(n\right)}^{\left(n_{0}\right)}$ is explicitly computed by inverting Eq. **10**:

[11] $$\begin{array}{c} {\tilde{R}}_{\left(n\right)}^{\left(n_{0}\right)}=a_{\left(n\right)}-F_{\left(n\right)}-\sum_{s=0}^{n-n_{0}-1}K_{\left(s+\frac{1}{2}\right)}v_{\left(n-s-\frac{1}{2}\right)}\Delta t. \end{array}$$

For $n=n_{0}$, we let ${\tilde{R}}_{\left(n\right)}^{\left(n\right)}=a_{\left(n\right)}-F_{\left(n\right)}$. The time-averaged estimator of $\left\langle R_{\left(k\right)}v_{\left(0\right)}\right\rangle$ can be written as $\zeta_{k}=\frac{1}{N_{k}}\sum_{n_{0}=0}^{N_{k}-1}{\tilde{R}}_{\left(n_{0}+k\right)}^{\left(n_{0}\right)}v_{\left(n_{0}\right)}$, where $N_{k}=N-k$. Note that $\zeta_{0}$ only depends on the force fields while $\zeta_{k}$ also depends on the memory kernel for k>0. It is not recommended, for numerical stability, to train F by imposing the orthogonality condition $\zeta_{0}=0$ directly. We recommended, instead, to train F by minimizing the noise within a maximum-likelihood perspective, and further decouple the training of F and K for stability and efficiency. To achieve these goals, we first define the constrained optimization problem:

[12] $$\begin{array}{c} \begin{array}{c} \underset{F^{\theta },K^{\theta }}{\text{minimize}}\&\underset{\kappa ∼\pi_{\kappa }}{E}\sum_{n=m_{K}}^{N-1}{\left|{\tilde{R}}_{\left(n\right)}^{\left(0\right)}\right|}^{2}\\ \text{subject to}\;\&\underset{\kappa ∼\pi_{\kappa }}{E}\zeta_{k}=0,\;\;k\in \left[1,m_{K}\right]. \end{array} \end{array}$$

Here, $\pi_{\kappa }$ denotes an ensemble of κ trajectories. The ensemble average $E_{\kappa ∼\pi_{\kappa }}$ is not necessary when κ is ergodic and sufficiently long. But in practice averaging over multiple finite-size trajectories is preferred. $m_{K}$ is the finite memory cutoff of K. $F^{\theta }$ and $K^{\theta }$ are the parameters of F and K, respectively. F can be any differentiable parameterized function, including neural networks. Eq. **12** should be transformed into an unconstrained problem in practical applications. Notice that the constraint $E_{\kappa ∼\pi_{\kappa }}\zeta_{k}=0$ can be written equivalently as

[13] $$\begin{array}{c} \begin{array}{cc} \underset{\kappa ∼\pi_{\kappa }}{E}\sum_{n_{0}=0}^{N_{k}-1} & \left(a_{\left(n_{0}+k\right)}-F_{\left(n_{0}+k\right)}\right)v_{\left(n_{0}\right)}\\ & =\underset{\kappa ∼\pi_{\kappa }}{E}\sum_{s=0}^{k-1}K_{\left(s+\frac{1}{2}\right)}\Delta t\sum_{n_{0}=0}^{N_{k}-1}v_{\left(n_{0}+k-s-\frac{1}{2}\right)}v_{\left(n_{0}\right)}. \end{array} \end{array}$$

Considering $k\in [1,m_{K}]$, Eq. **13** can be written in matrix form as Y=CK. Y and K are vectors of length $m_{K}$. C is a $m_{K}\times m_{K}$ lower triangular matrix. The left-hand side of Eq. **13** is the k-th entry of Y. The j-th entry of K is $K_{j}=K_{\left(j-\frac{1}{2}\right)}\Delta t$. And $C_{\mathrm{kj}}=E_{\kappa ∼\pi_{\kappa }}\sum_{n_{0}=0}^{N_{k}-1}v_{\left(n_{0}+k-j+\frac{1}{2}\right)}v_{\left(n_{0}\right)}$ when $m_{K}\geq k\geq j\geq 1$. Hence, the least-square solution of Eq. **13** can be written as $K=\text{Inv}\left(C^{T}C\right)C^{T}Y$. Inv is the pseudoinverse operator computed from single-value decomposition with a cutoff ratio to avoid numerical instability.

We are then able to approach the solution of Eq. **12** practically by interleaving $n^{\mathrm{GD}}\geq 1$ unconstrained optimization steps toward

[14] $$\begin{array}{c} \underset{F^{\theta }}{\text{minimize}}\underset{\kappa ∼\pi_{\kappa }}{E}\sum_{n=m_{K}}^{N-1}{\left|{\tilde{R}}_{\left(n\right)}^{\left(0\right)}\right|}^{2} \end{array}$$

with one iteration of

[15] $$\begin{array}{c} \begin{array}{cc} K & \to \left(1-\epsilon \right)K+\epsilon \text{Inv}\left(C^{T}C\right)C^{T}Y,\\ K & \to K-K_{m_{K}}1_{m_{K}}. \end{array} \end{array}$$

The parameter ϵ∈(0,1) should be small enough for stability. In this work, we use ϵ=0.01. The second step in Eq. **15** forces $K_{m_{K}}=0$ over the course of training.

### Training of the GAR Model.

In the previous step, the noise $R_{\left(n\right)}={\tilde{R}}_{\left(n\right)}^{\left(0\right)}$ is extracted from $a_{\left(n\right)}$. Then, one can establish a GAR model with $R_{\left(n\right)}$ as data. The GAR parameters include the linear coefficients $\phi =(\phi_{\left(1\right)},\cdots ,\phi_{\left(k\right)})$ and the parameters $\left\{\mu^{\theta },\sigma^{\theta }\right\}$ of the neural network. We define the maximum likelihood loss function

[16] $$\begin{array}{c} L^{\mathrm{GAR}}=\underset{\kappa ∼\pi_{\kappa }}{E}\sum_{n}\ln\sigma_{\left(n\right)}^{2}+\frac{{\left(R_{\left(n\right)}-\sum_{k=1}^{m_{A}}\phi_{\left(k\right)}R_{\left(n-k\right)}-\mu_{\left(n\right)}\right)}^{2}}{\sigma_{\left(n\right)}^{2}}. \end{array}$$

It is not recommended to minimize $L^{\mathrm{GAR}}$ directly with respect to all the parameters without constraints. Overfitting the data should be avoided for the long-term stationarity of the GAR model. This is crucially important for simulating AIGLE at or above the μs scale, much longer than the picosecond/nanosecond duration of the MD trajectories. So, we harness the GAR model by imposing on ϕ the constraint that they should satisfy the Yule–Walker equation. For a given CV trajectory, let $\lambda_{\left(k\right)}$ be the estimator of the noise ACF, given by $\lambda_{\left(k\right)}=\frac{1}{N_{k}-m_{A}}\sum_{n=m_{A}}^{N_{k}-1}R_{\left(n+k\right)}R_{\left(n\right)}$. Let the vector Λ be $\Lambda =\underset{\kappa ∼\pi_{\kappa }}{E}\left(\lambda_{\left(1\right)},\cdots ,\lambda_{\left(m_{A}\right)}\right)$. Let the $m_{A}\times m_{A}$ matrix M be $M_{\mathrm{jk}}=\underset{\kappa ∼\pi_{\kappa }}{E}\lambda_{\left(|j-k|\right)}$. The Yule–Walker equation for a standard AR($m_{A}$) model is Λ=Mϕ, the least square solution of which can be written as $\phi^{\mathrm{YW}}=\text{Inv}\left(M^{T}M\right)M^{T}\Lambda$. Using the Yule–Walker solution as a constraint, we optimize the GAR model by interleaving $n^{\mathrm{GD}}$ unconstrained optimization steps toward

[17] $$\begin{array}{c} \underset{\mu^{\theta },\sigma^{\theta }}{\text{minimize}}\;\;L^{\mathrm{GAR}} \end{array}$$

with one iteration of

[18] $$\begin{array}{c} \phi \to \left(1-\epsilon \right)\phi +\epsilon \text{Inv}\left(M^{T}M\right)M^{T}\Lambda . \end{array}$$

Although in the formal presentation, the training of GAR is done after the training of the first step, in practice one can train GAR on the fly to simplify the implementation.

### Incorporation of Near-Equilibrium Data.

In this step, we deal with additional datasets that violate detailed balance. We fix the memory kernel and the GAR model obtained for thermal equilibrium, assuming that they are approximately the same in near-equilibrium situations. The optimization task is simply

[19] $$\begin{array}{c} \underset{F^{\theta }}{\text{minimize}}\underset{\kappa ∼\pi_{\kappa }}{E}\sum_{n=m_{K}}^{N-1}{\left|{\tilde{R}}_{\left(n\right)}^{\left(0\right)}\right|}^{2} \end{array}$$

for the extended dataset. Here $F^{\theta }$ may include the parameters of the external driving forces.

## Supplementary Material

Appendix 01 (PDF)

## Data Availability

The DP model and a minimal implementation of AIGLE are publicly available on Github (93).
