# Supplementary material for: Ab initio generalized Langevin equation
Source: Proc Natl Acad Sci U S A. 2024 Mar 29;121(14):e2308668121. doi: 10.1073/pnas.2308668121 (PMC10998567; doi:10.1073/pnas.2308668121)
Supplement: Supplementary file 1 — Appendix 01 (PDF) [file pnas.2308668121.sapp.pdf]

Supporting Information for

Ab Initio Generalized Langevin Equation

Pinchen Xie, Roberto Car, Weinan E

Pinchen Xie.
E-mail: pinchenx@math.princeton.com

This PDF file includes:

Supporting text
Figs. S1 to S5
SI References

Supporting Information Text

1. Validation with a solvable model

We study a toy model with a known memory kernel (1) in the MZ framework under thermal equilibrium conditions. The model consists of a one-dimensional harmonic chain of $N+1$ identical particles of mass m. The chain has one free and one fixed end. Let $\{z_i\}_{i\in[0,N]}$ and $\{p_i\}_{i\in[0,N]}$ be the coordinate and momentum of the particles in the harmonic chain. Let $z_0 = 0$ and $p_0 = 0$. The Hamiltonian of the system is

$$H(\{p_i\},\{z_i\}) = \sum_{i=0}^{N-1} \frac{p_{i+1}^2}{2m} + \frac{1}{2}\tilde{\omega}^2(z_i - z_{i+1} - a)^2. \quad [1]$$

The parameters of the system we simulate are $N = 100$, $m = k_B T = \tilde{\omega} = 1$. x_N is connected to a Langevin thermostat with damping time $t_d = 10$, representing a gas/solid interface. The CV x is chosen to be the displacement of the free end of the chain from its equilibrium position, i.e., $x = x_N$. We use a MD time step $\delta t = 0.1$ and obtain 200 trajectories of x, each lasting $t_{MD} = 2000$ after initial equilibration. The trajectories are further coarse-grained with a time step $\Delta t = 4\delta t$ by sampling the MD data every 4 MD time steps. The GLE ansatz for this system can be written as

$$a_{(n)} = -\omega_0^2 x_{(n)} + \sum_{s=0}^{n-1} K_{(s+\frac{1}{2})} v_{(n-s-\frac{1}{2})}\Delta t + R_{(n)}. \quad [2]$$

The neural network part of the corresponding GAR model is a feed-forward neural network with two hidden layers (size=10). The neural network only outputs $\mu_{(n)}$ in the GAR model. The $\sigma_{(n)}$ in the GAR model is parameterized directly by one scalar variable since its history dependence is unnecessary for this case study. The finite memory cutoff is taken to be $m_K = 50$ for the memory kernel and $m_A = 25$ for the GAR model. The training is done with the Adam optimizer implemented in PyTorch (2) with the default setting and learning rate of 0.001. Convergence is reached with approximately 3000 iterations. For each iteration, 50 trajectories of the CV are randomly chosen for optimization. The parameters ϵ for the iterative update of memory kernel and the Yule-Walker coefficient are all 0.01. The interval n^{GD} is 10. The optimized ω_0 is 0.09. The optimized standard deviation of the white noise is $\sigma_{(n)} = 0.721$. The colored noise R and the white noise w are both distributed normally on the dataset with negligible off-centering. Non-stationarity is not detected numerically for the GAR model.

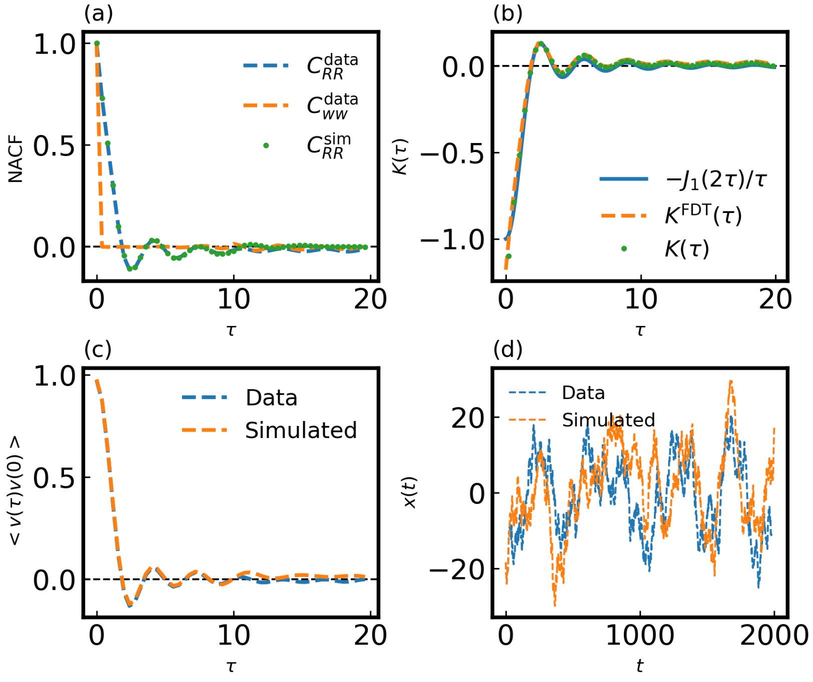

Fig. S1. (a) NACF of the noise. $C_{RR}^{\mathrm{data}}(\tau) = \langle R(t_0+\tau)R(t_0)\rangle/\langle R(t_0)^2\rangle$ and $C_{RR}^{\mathrm{sim}}(\tau) = \langle R(t_0+\tau)R(t_0)\rangle/\langle R(t_0)^2\rangle$ are averaged over the dataset and the simulated trajectories, respectively. $C_{ww}^{\mathrm{data}}(\tau) = \langle w(t_0+\tau)w(t_0)\rangle/\langle w(t_0)^2\rangle$ is the NACF of the residual noise from the dataset. (b) The optimized memory kernel $K(\tau)$ compared to the theoretical one and the 2FDT prediction $K^{\mathrm{FDT}}(\tau = n\Delta t) = -\beta\langle R_{(n)}R_{(0)}\rangle$. (c) The velocity autocorrelation function. (d) Comparison between an MD trajectory and a GLE trajectory with the same initial conditions.

The outcome of the training is reported in Fig. S1. The GAR model successfully reduces w to almost white noise with delta-like normalized autocorrelation function (NACF), as shown in Fig. S1(a). An obvious serial correlation would be present if we used an AR model instead. Both R and w are normally distributed on the dataset. Fig. S1(b) shows that the optimized $K(\tau)$ obeys 2FDT and agrees well with the theoretical result for an isolated infinite chain, $K^{\mathrm{MZ}}(\tau) = -J_1(2\tau)/\tau$, where J_1

is a Bessel function of the first kind. The discrepancy near $\tau = 0$ is due to the inclusion of white noise from the Langevin thermostat. Fig. S1(c) compares the velocity autocorrelation from the MD dataset and the AIGLE simulation. They display minor disagreement for $\tau > 10$. This is the error caused by substituting $w_{(n)}$ with the ideal white noise generator when propagating AIGLE. The same effect can be seen in Fig. S1(a) on the slight discrepancy between $C_{RR}^{\rm data}$ and $C_{RR}^{\rm sim}$. We also compare a simulated AIGLE trajectory and an MD trajectory with the same initial conditions in Fig. S1(d). They display statistically equivalent behavior of stochastic harmonic oscillators.

2. Continuous-time on-lattice GLE

A. Variational approach for memory kernel. Following the notation used in the main text, the GLE for $\boldsymbol{x}_i = (x_{i1}, \cdots, x_{id_s})^T$ is

$$M\boldsymbol{a}(t) = \boldsymbol{\mathcal{F}}(t) + \int_0^t dsMK(s)\boldsymbol{v}(t-s) + \boldsymbol{R}(t). \quad [3]$$

We recall the orthogonality condition $\langle \boldsymbol{R}(t)\boldsymbol{v}^T(0)\rangle = 0$, and the 2FDT

$$\langle \boldsymbol{v}(0)\boldsymbol{v}^T(0)\rangle K^T(s) = -\langle (M^{-1}\boldsymbol{R})(0)(M^{-1}\boldsymbol{R})^T(s)\rangle, \quad [4]$$

for the equilibrium ensemble. We start by formulating a variational principle for the most general memory kernel, i.e., a kernel $K(s) = \{K_{i\alpha}^{j\beta}(s)\}$ represented by a dense tensor. Using Eq. (3), the orthogonality tensor defined by $\Omega_{i\alpha}^{j\beta}(t) = \langle R_{i\alpha}(t)v_{j\beta}(0)\rangle$, can be written as

$$\Omega_{i\alpha}^{j\beta}(t) = Q_{i\alpha}^{j\beta}(t) - \int_0^t ds(MK)_{i\alpha}^{k\gamma}(s)C_{k\gamma}^{j\beta}(t-s), \quad [5]$$

where the Einstein notation for repeated indices is used, $Q_{i\alpha}^{j\beta}(t) = \langle (Ma-\mathcal{F})_{i\alpha}(t)v_{j\beta}(0)\rangle$, and $C_{k\gamma}^{j\beta}(t-s) = \langle v_{k\gamma}(t-s)v_{j\beta}(0)\Theta(t-s)\rangle$, with Θ the Heaviside step function ($\Theta(x) = 1$ for $x > 0$, $\Theta(x) = 0$ otherwise). In general, the deterministic force $\mathcal{F}$ can be defined by force matching. Assuming that the exact $\mathcal{F}$ is known, the diagonal element of the orthogonality tensor at $t = 0$ vanishes, i.e., $\Omega_{i\alpha}^{i\alpha}(0) = Q_{i\alpha}^{i\alpha}(0) = 0$

Given a canonical ensemble of trajectories and the corresponding exact $\mathcal{F}$, the orthogonality condition implies that the exact memory kernel K minimizes the canonical orthogonality loss functional $\tilde{\mathcal{L}}[K]$, defined by

$$\tilde{\mathcal{L}}[K] = \int_0^\infty \sum_{i,j,\alpha,\beta} |\Omega_{i\alpha}^{j\beta}(t)|^2 dt. \quad [6]$$

By exploiting locality in space and finite memory time, we approximate $\tilde{\mathcal{L}}$ with $\mathcal{L}$ given by

$$\mathcal{L}[K](t_K) = \int_0^{t_K} \sum_{i,j,\alpha,\beta} |\Omega_{i\alpha}^{j\beta}(t)|^2 \Theta(d_c - d_{ij})dt, \quad [7]$$

where t_K is the cutoff time of the memory, d_c is the cutoff length of the correlation in space, and d_{ij} is the graph geodesic distance between sites i and j, given the adjacency matrix A of the lattice. We require $K(s) = 0$ for $s > t_K$. A bulk crystalline system, under equilibrium conditions, has translational symmetry, and the complexity of evaluating $\mathcal{L}[K](t_K)$ is limited by d_c and t_K, regardless of the size of the lattice. Then, the optimal $K = K^*$ for given d_c and t_K is found by minimization, i.e.,

$$K^* = \mathrm{argmin}_K \mathcal{L}[K](t_K) \quad [8]$$

B. Local kernel approximation. The approximation introduced in the previous subsection is still excessively complicated for use in practical applications. Here, we make a further important simplification, valid when the velocity correlations of the CVs in the extensive coarse-grained lattice dynamics are negligible beyond nearest neighbors. Then, $1 < d_c < 2$, and the functional in Eq. (7) becomes

$$\mathcal{L}[K](t_K) = \int_0^{t_K} \sum_{i,j,\alpha,\beta} |\Omega_{i\alpha}^{j\beta}(t)|^2 (\delta_{ij} + A_{ij})dt. \quad [9]$$

We also assume that $M_{i\alpha}^{j\beta} = \delta_{ij}\delta_{\alpha\beta}M_\alpha$ has been determined from the data by applying the equipartition theorem to the kinetic energy of the CVs. The local kernel approximation for K, adopted in the main text, assumes that only the on-site elements $K_{i\alpha}^{i\beta}(s)$ can be nonzero. Intuitively, one expects that the main contribution to memory should be on-site. In practice, the local kernel approximation is justified when the corresponding coarse-grained dynamics reproduces with sufficient accuracy the dynamics inferred from the underlying microscopic Hamiltonian. We notice that the local kernel approximation would be compatible with a less restrictive choice of the spatial cutoff d_c in Eq. (7). In this way, the memory kernel will still be on-site but will depend effectively on longer range correlations between the CVs. Since, by translational symmetry, $K_{i\alpha}^{i\beta}(s)$ does not depend on the site index i, we drop this index in the following, and use the notation $K_\alpha^\beta(s)$ for K, and $\Omega_{\alpha,\beta}^0$ for $\Omega_{i\alpha}^{i\beta}$ For the same reason, $\Omega_{i\alpha}^{j\beta}$ should depend only on the relative displacement $\boldsymbol{\eta}_{ij}$ connecting sites i and j in the lattice. Let $\{\boldsymbol{\eta}_\iota | \iota = 1, \cdots, d_{\rm nn}\}$ be the set of different lattice vectors connecting nearest neighbor sites, and let η_0 be the null vector. Using

the label $i_{[\iota]}$ to indicate a site displaced from site-i by $\boldsymbol{\eta}_\iota$, we further simplify the notation for $\Omega_{i\alpha}^{i_{[\iota]}\beta}$ to $\Omega^\iota_{\alpha,\beta}$. Then, for arbitrary i and j, we have $\Omega_{i\alpha}^{j\beta}(\delta_{ij}+A_{ij}) \in \{\Omega^\iota_{\alpha,\beta} | \iota = 0, \cdots, d_{\mathrm{nn}}\}$. Then, Eq. (9) becomes

$$\mathcal{L}[K](t_K) = L \int_0^{t_K} \sum_{\alpha,\beta} \sum_{\iota=0}^{d_{\mathrm{nn}}} |\Omega^\iota_{\alpha,\beta}(t)|^2 dt. \tag{10}$$

Here, $\Omega^\iota_{\alpha,\beta}(t)$ is given by

$$\Omega^\iota_{\alpha,\beta}(t) = Q^\iota_{\alpha,\beta}(t) - \int_0^t ds M_\alpha \sum_\gamma K^\gamma_\alpha(s) C^\iota_{\gamma,\beta}(t-s). \tag{11}$$

In Eq. (11), $Q^\iota_{\alpha,\beta}(t) = \frac{1}{L}\sum_i \langle (Ma - \mathcal{F})_{i\alpha}(t) v_{i_{[\iota]}\beta}(0)\rangle$, and $C^\iota_{\gamma,\beta}(t-s) = \frac{1}{L}\sum_i \langle v_{i\gamma}(t-s) v_{i_{[\iota]}\beta}(0)\Theta(t-s)\rangle$ are directly calculated from the data. Writing this equation in matrix form (with respect to the indices α and β) leads to

$$\Omega^\iota(t) = Q^\iota(t) - \int_0^t ds M K(s) C^\iota(t-s). \tag{12}$$

Here all matrices are of size $d_s \times d_s$. Taking the functional derivative of $\mathcal{L}[K](t_K)$ with respect to $M_\alpha K^\gamma_\alpha(s)$ one obtains

$$\frac{\delta \mathcal{L}[K](t_K)}{\delta M_\alpha K^\gamma_\alpha}(s) - 2L \int_s^{t_K} dt \sum_\beta \sum_{\iota=0}^{d_{\mathrm{nn}}} \Omega^\iota_{\alpha,\beta}(t) C^\iota_{\gamma,\beta}(t-s) \tag{13}$$

Putting Eq. (13) in matrix form and inserting Eq. (12) leads to

$$\begin{aligned} \frac{\delta \mathcal{L}[K](t_K)}{\delta (MK)}(s) &= -2L \sum_{\iota=0}^{d_{\mathrm{nn}}} \int_s^{t_K} dt \Big(Q^\iota(t) (C^\iota)^T(t-s) \\ &\quad - \int_0^t dr M K(r) C^\iota(t-r)(C^\iota)^T(t-s) \Big) \\ &= -2L \sum_{\iota=0}^{d_{\mathrm{nn}}} \int_0^{t_K} dt \Big(Q^\iota(t) (C^\iota)^T(t-s) \\ &\quad - \int_0^{t_K} dr M K(r) C^\iota(t-r)(C^\iota)^T(t-s) \Big) \end{aligned} \tag{14}$$

The second equality holds because by definition $C^\iota(x) = 0$ for $x < 0$.

Finally, K^* is the least square solution of $\frac{\delta \mathcal{L}[K](t_K)}{\delta (MK)}(s) = 0$, i.e.,

$$\begin{aligned} &\sum_{\iota=0}^{d_{\mathrm{nn}}} \int_0^{t_K} dr M K(r) \int_0^{t_K} dt C^\iota(t-r)(C^\iota)^T(t-s) \\ &= \sum_{\iota=0}^{d_{\mathrm{nn}}} \int_0^{t_K} dt Q^\iota(t) (C^\iota)^T(t-s). \end{aligned} \tag{15}$$

In the continuous-time formulation of the GLE it is not evident how one could find the solution K^* via a standard least-square algorithm. This will become evident in the next section where we use the discrete-time formulation of the GLE recommended in practical implementations.

3. Discrete-time on-lattice AIGLE

A. Formulation. The discrete-time GLE is an approximation to the continuous-time GLE. Here we follow the same leap-frog strategy adopted for univariant AIGLE. The discrete-time on-lattice AIGLE takes the form

$$M\boldsymbol{a}_{(n)} = \boldsymbol{\mathcal{F}}_{(n)} + \sum_{l=0}^{n-1} M K_{(l+\frac{1}{2})} \boldsymbol{v}_{(n-l-\frac{1}{2})} \Delta t + \boldsymbol{R}_{(n)}. \tag{16}$$

Here Δt is the time step of integration, n is an integer, and $t = n\Delta t$ is the current time. We use $f_{(n)}$ as an abbreviation for any time-dependent function $f(n\Delta t)$. The integration scheme for the GLE is given by

$$\begin{aligned} \boldsymbol{v}_{(n+\frac{1}{2})} &= \boldsymbol{v}_{(n-\frac{1}{2})} + \boldsymbol{a}_{(n)} \Delta t, \\ \boldsymbol{x}_{(n+1)} &= \boldsymbol{x}_{(n)} + \boldsymbol{v}_{(n+\frac{1}{2})} \Delta t. \end{aligned} \tag{17}$$

We call $\{n\Delta t|n \in \mathbb{Z}\}$ the integer grid and $\{(n-\frac{1}{2})\Delta t|n \in \mathbb{Z}\}$ the shifted grid. The trajectory data $x_{(n)}$ are given on the integer grid. $\boldsymbol{v}_{(n-\frac{1}{2})}$ and $\boldsymbol{a}_{(n)}$ can are computed directly from Eq. (17). $\boldsymbol{v}_{(n)}$ on the integer grid is subsequently intepolated as $\boldsymbol{v}_{(n)} = (\boldsymbol{v}_{(n+\frac{1}{2})} + \boldsymbol{v}_{(n-\frac{1}{2})})/2$.

Let $t_K = m_K \Delta t$ be the cutoff of the memory time. Then, $K_{(l+\frac{1}{2})} = 0$ for $l \geq m_K$. The quantity $Q^{\iota}_{\alpha,\beta}(n\Delta t)$ of the continuous-time formulation becomes $Q^{\iota}_{\alpha,\beta,(n)} = \frac{1}{L}\sum_i \langle (Ma - \mathcal{F})_{i\alpha,(n)} v_{i_{[\iota]}\beta,(0)} \rangle$ in the discrete time representation, and $C^{\iota}_{\gamma,\beta}((n-l+\frac{1}{2})\Delta t)$ becomes $C^{\iota}_{\gamma,\beta,(n-l)} = \frac{1}{L}\sum_i \langle v_{i\gamma,(n-l+\frac{1}{2})} v_{i_{[\iota]}\beta,(0)} \Theta(n-l+\frac{1}{2}) \rangle$. To avoid messy indexing, in the following we make all the indices labelling a local order parameter component implicit. With this simplified notation $Q^{\iota}_{(n)}$ stands for the $d_s \times d_s$ matrix of which the (α,β)-entry is $Q^{\iota}_{\alpha,\beta,(n)}$, $C^{\iota}_{(n-l)}$ stands for the $d_s \times d_s$ matrix of which the (α,β)-entry is $C^{\iota}_{\alpha,\beta,(n-l)}$, $K_{(l+\frac{1}{2})}$ stands for the $d_s \times d_s$ matrix of which the (α,β)-entry is $K^{\beta}_{\alpha,(l+\frac{1}{2})}$.

B. Memory kernel. The results of Sec. 2 can be translated straightforwardly into discrete-time notation. Thus, we go directly to the local kernel approximation and show how the optimal memory kernel can be obtained in practice.

Eq. (10) becomes

$$\mathcal{L}[K](t_K) = L\sum_{n=1}^{m_K}\sum_{\alpha,\beta}\sum_{\iota=0}^{d_{\mathrm{nn}}} |\Omega^{\iota}_{\alpha,\beta,(n)}|^2 \Delta t. \tag{18}$$

The matrix $\Omega^{\iota}_{(n)} = (\Omega^{\iota}_{\alpha,\beta,(n)})_{1\leq\alpha,\beta\leq d_s}$ is given by

$$\Omega^{\iota}_{(n)} = Q^{\iota}_{(n)} - \sum_{l=0}^{n-1} MK_{(l+\frac{1}{2})} C^{\iota}_{(n-l-1)} \Delta t, \tag{19}$$

with $n \geq 1$. $\Omega^{\iota}_{(0)} = Q^{\iota}_{(0)}$ is excluded from Eq. (18) because it does not depend on the memory kernel.

Similar to the derivation of Eq. (15) from Eq. (10), the optimal condition for the memory kernel is derived from Eq. (18), giving

$$\sum_{l=0}^{m_K-1} \underbrace{MK_{(l+\frac{1}{2})}\Delta t}_{U_l} \underbrace{\sum_{\iota=0}^{d_{\mathrm{nn}}}\sum_{n=0}^{m_K-1} C^{\iota}_{(n-l)} (C^{\iota}_{(n-k)})^T \Delta t}_{V_{l,k}} = \underbrace{\sum_{\iota=0}^{d_{\mathrm{nn}}}\sum_{n=0}^{m_K-1} Q^{\iota}_{(n+1)} (C^{\iota}_{(n-k)})^T \Delta t}_{W_k}. \tag{20}$$

To make implementations with standard linear algebra routines evident, in Eq. (20) we further define the $d_s \times d_s$ matrices U_l, $V_{l,k}$ and W_k. $V_{l,k}$ and W_k are obtained directly from the data, while U_l is to be found with a tensor least square routine.

For the special case for which $d_s = 1$, U_l, $V_{l,k}$ and W_k are just scalars, and we define the vector $U = (U_0, \cdots, U_{m_K-1})$, the matrix $V = (V_{l,k})_{0\leq l\leq m_K-1; 0\leq k\leq m_K-1}$, and the vector $W = (W_0, \cdots, W_{m_K-1})$. Note that V is positive semi-definite. This is the simplest case of local kernel approximation, for which $U = V^{-1}W$.

C. Noise generator. Under local kernel approximation, the memory kernel is one-body, but the time evolution of R can not be reduced to a one-body equation of motion. Then, the univariant GAR model should be generalized to the multi-dimensional case. Let $\boldsymbol{R}_{i,(n)} = (R_{i1,(n)}, \cdots, R_{id_s,(n)})$ be the d_s-dimensional noise acting on site-i at time step n. Let n_A be any positive integer. Let $(\phi_{\alpha,k})_{1\leq\alpha\leq d_s, 1\leq k\leq n_A}$ be a $d_s \times n_A$ real matrix . Assuming translational symmetry, the multi-dimensional GAR model for predicting $\boldsymbol{R}_{i,(n)}$ from the history of noise is given by

$$\boldsymbol{R}_{i\alpha,(n)} = \sum_{k=1}^{m_A} \phi_{\alpha,k} R_{i\alpha,(n-k)} + \sum_{j\in\mathcal{N}(i)} \mu^j_{i\alpha,(n)} + \sigma_{i\alpha} w_{i\alpha,(n)} \tag{21}$$

and

$$\mu^j_{i\alpha,(n)} = \mu^j_{i\alpha}\big(R_{j\alpha,(n-1)}, \cdots, R_{j\alpha,(n-m_A)}\big). \tag{22}$$

Here, $\phi_{\alpha,k}$ are the Yule-Walker linear autoregressive parameters (3) for $R_{i\alpha,(n)}$ as a time series with respect to n. $\sigma_{i\alpha} = \sigma_\alpha$ is a scalar parameter. In principle, $\sigma_{i\alpha}$ could be a function of the history of $R_{i\alpha,(n)}$, but in all the cases we studied we found that assuming a scalar $\sigma_{i\alpha}$ was sufficient for accurate autoregression, which is the choice that we adopt in the following. $w_{i\alpha,(n)}$ is a standard Gaussian white noise, independently sampled for each i, α and n. $\mathcal{N}(i)$ is the neighborhood of site-i. Typically, we can let $\mathcal{N}(i)$ includes site-i and all site-j satisfying $A_{ij} = 1$. It is also feasible to include second nearest neighbor beyond $A_{ij} = 1$ in $\mathcal{N}(i)$. $\mu^j_{i\alpha}$ is a one-body function taking as input the history of $R_{j\alpha,(n)}$ as a time series with respect to n. In ab initio GLE, we use a collection of small feed-forward neural networks to represent $(\mu^j_{i\alpha})_{1\leq\alpha\leq d_s}$. The size of the collection can be reduced when system symmetry is taken into consideration. For a translationall-invariant lattice, $(\mu^j_{i\alpha})$ is decided by the separation between site-i and site-j. So the complexity of the GAR model does not grow with the lattice size.

Training of the multi-dimensional GAR model is similar to that of the univariant GAR model. With a given K^*, we extract the time series $\boldsymbol{R}_{(n)}$ from

$$\boldsymbol{R}_{(n)} = M\boldsymbol{a}_{(n)} - \boldsymbol{\mathcal{F}}_{(n)} - \sum_{s=0}^{n-1} MK^*_{(s+\frac{1}{2})} \boldsymbol{v}_{(n-s-\frac{1}{2})} \Delta t. \tag{23}$$

Using $\{\boldsymbol{R}_{(n)}\}$ as data, we first determine the Yule-Walker solution for $\phi_{\alpha,k}$ using a least square routine. Then, we train the parameters in $(\mu^j_{i\alpha})_{1\leq\alpha\leq d_s}$ and $(\sigma_\alpha)_{1<\alpha<d_s}$ using the maximum likelihood loss function

$$\Gamma = \sum_{i\alpha}\sum_{n}\log\sigma_\alpha^2 + \frac{\left(R_{i\alpha,(n)} - \sum_{k=1}^{m_A}\phi_{\alpha,k}R_{i\alpha,(n-k)} - \sum_{j\in\mathcal{N}(i)}\mu^j_{i\alpha,(n)}\right)^2}{\sigma_\alpha^2} \qquad [24]$$

After training, one can run the GAR model independently, and calculate the noise ACF $\Upsilon_{(n)} = \langle(\boldsymbol{M}^{-1}\boldsymbol{R})_{(0)}(\boldsymbol{M}^{-1}\boldsymbol{R})^T_{(n)}\rangle_{\text{GAR}}$. The subscript "GAR" indicates that the ensemble average is calculated over trajectories generated by the GAR simulation. Then, the ACF of the noise can be compared with $\langle\boldsymbol{v}_{(0)}\boldsymbol{v}^T_{(0)}\rangle K^{*T}_{(n)}$ to verify that the 2FDT is satisfied. For that purpose, the equal time velocity correlation matrix $\mathcal{G} = \langle\boldsymbol{v}_{(0)}\boldsymbol{v}^T_{(0)}\rangle$ is extracted from the data. A small deviation of $\Upsilon_{(n)}$ from $\mathcal{G}K^{*T}_{(n)}$ should be expected, reflecting a slight violation of the 2FDT derived from the data. This is an inevitable consequence of the approximation made for the memory kernel, i.e., the local kernel approximation. Thus, the equal-time velocity correlation matrix from the AIGLE simulation will deviate slightly from the corresponding matrix from the MD data. One may try to reduce this error by modifying the memory kernel to enforce the 2FDT directly. We call K_*, a modified memory kernel that minimizes the target function

$$\mathcal{L}^{\text{FDT}}[K] = L\sum_{n=1}^{m_K}\sum_{\alpha,\beta}\sum_{\iota=0}^{d_{\text{nn}}}\left(\sum_{\gamma}\mathcal{G}^\iota_{\alpha,\gamma}K^\gamma_{\beta,(n)} - \Upsilon^\iota_{\alpha,\beta,(n)}\right)^2, \qquad [25]$$

where we follow the conventions of subsection A, i.e., $\mathcal{G}^\iota_{\alpha,\gamma} = \frac{1}{L}\sum_i \mathcal{G}^{i_{[\iota]}\gamma}_{i\alpha}$, and $\Upsilon^\iota_{\alpha,\beta,(n)} = \frac{1}{L}\sum_i \Upsilon^{i_{[\iota]}\beta}_{i\alpha,(n)}$. Thus, $K_* = \text{argmin}_K\mathcal{L}^{\text{FDT}}[K]$, and can be found straightforwardly with a least square scheme. K_* is different from K^*, defined in subsection B. In practice, the difference between the two approximate kernels is mostly cosmetic. While K^* is more faithful to the data in terms of the time series regression, K_* is more faithful to the data in terms of the equal-time velocity correlation matrix. In practice, the two approaches produce minor quantitative differences, but are equivalent in terms of physical accuracy.

4. Details of modeling domain wall motion as a virtual particle

A. DFT-based atomistic models. Our atomistic model for $PbTiO_3$ is based on the methodology developed in Refs. (4–7). The DP model for representing the potential energy surface is trained on DFT data with the SCAN meta-GGA functional (8). The dataset is collected through the active learning procedure implemented in the DPGEN code (9, 10). The data are labeled by Quantum ESPRESSO (11) with norm-conserving pseudo-potentials (12) including semi-core states. An early version of the current DP model was introduced in Ref. (7). It was trained with configurations without domain walls and used to study the ferroelectric phase transition in bulk $PbTiO_3$ with atomistic simulations. The model predicted thermodynamic and ferroelectric properties of $PbTiO_3$ in close agreement with experimental results, and provided unique insight into the microscopic mechanism driving the phase transition. To train the current DP model we follow the same numerical protocol adopted in Ref. (7), where the full technical details can be found. The final dataset for the current DP model contains configurations without domain walls and with two twin 90° domain walls or two twin 180° domain walls. 1276 configurations with no domain walls are collected with the active learning procedure of Ref. (7) in the temperature interval [300K, 1200K] and the pressure interval [0, 10^5Pa]. 494 configurations with domain walls are collected with the same active learning procedure in the temperature interval $[100K, 600K]$ and the pressure interval [0, 10^5Pa]. The largest supercell with domain wall configurations contains 54 elementary cells, corresponding to 270 atoms. We use a spatial cutoff radius of 8Å for the DP model. This is larger than in typical DP models but is necessary to capture long-range interactions in the presence of two domain walls with periodic boundary conditions.

Fig. S2 plots the nearly Gaussian error distribution of the DP model against the training set. The root-mean-square error (RMSE) of the energy is 1meV/atom for configurations without domain walls and 1.4meV/atom for the remaining ones. The RMSE of the Cartesian components of the forces is around 0.1eV/Å in all cases. The force error is slightly larger for the direction orthogonal to the domain walls, which is the x-axis for our data with 180° domain walls. The DP model is also validated on a small independent testing set, showing similar accuracy to its performance on the training set. We conclude that the DP model reproduces statistically the adiabatic potential energy surface of SCAN-DFT.

Next, we calculate the Berry-phase polarization change, relative to the centrosymmetric structure for all configurations without domain walls. The calculation is done with the Wannier90 code (13). The total polarization is the sum of electronic (Berry phase) contributions and ionic contributions (14). The dipole D of the simulation cell (cell dipole) is obtained by multiplying the total polarization by the volume of the cell. The Born charge (BC) associated with atom-i is the tensor $Z_{i,\alpha\beta} = \frac{\partial D_\alpha}{\partial x_{i,\beta}}$ defined as the change of D in direction α caused by the displacement of atom-i away from its equilibrium position in direction β. We adopt a linear approximation for the dependence of D_α on the atomic coordinates, given by $D_\alpha = \sum_i\sum_{\beta\in\{x,y,z\}}Z_{i,\alpha\beta}x_{i,\beta}$, which, in the manuscript, is referred to as the BC model. Due to space-group symmetry or negligible contribution, some degrees of freedom of the BC model can be eliminated. For $PbTiO_3$, the BCs of Pb and Ti atoms are conventionally approximated (15) by diagonal matrices $Z_{\text{Pb},\alpha\beta} = Z_{\text{Pb}}\delta_{\alpha\beta}$ and $Z_{\text{Ti},\alpha\beta} = Z_{\text{Ti}}\delta_{\alpha\beta}$, respectively. The BC of the O atom is also given a diagonal approximation, with values Z_{O1} for the Ti-O bond direction and Z_{O2} for the other

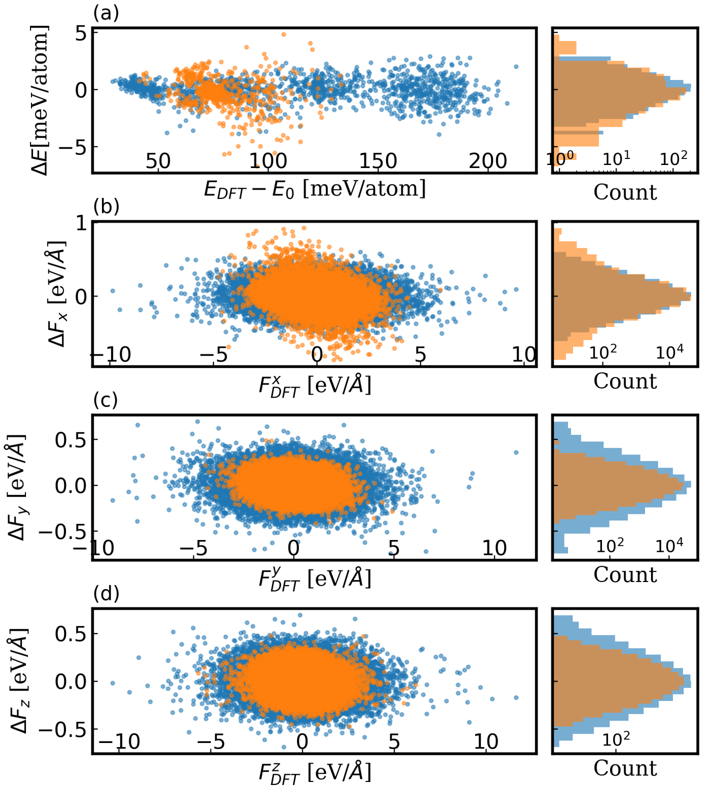

Fig. S2. Error distribution of the DP model on the training set. The blue color marks configurations without domain walls, and the orange color marks the rest. E_{DFT}, $F^{x,y,z}_{\mathrm{DFT}}$ are the energy and force labels of the data. E_0 is a constant. ΔE, $\Delta F_{x,y,z}$ are the difference between the model prediction and the data label.

two orthogonal directions. In the present study, the BC model is fitted to the cell dipole data with mean squared error loss, leading to $Z_{\mathrm{Pb}} = 3.7140e$, $Z_{\mathrm{Ti}} = 5.4897e$, $Z_{\mathrm{O1}} = -3.3551e$ and $Z_{\mathrm{O2}} = -2.9234e$. The RMSE of the cell dipole predicted by the resultant BC model is 2eÅ for a $3 \times 3 \times 3$ supercell. This corresponds to roughly a $2\mu\mathrm{C/cm}^2$ RMSE in polarization, much smaller than the $72\mu\mathrm{C/cm}^2$ total polarization of $PbTiO_3$ extracted from our room temperature simulations. The BC model constructed in this way is statistically more accurate at finite temperature than models using conventional Born effective charges calculated by perturbing the ground state equilibrium structure (16). Using the BC model so defined, the local electric dipole p_j associated with each Ti-centered elementary cell-j of $PbTiO_3$ is given by a weighted sum of the position of the atoms in the cell-j (17, 18). The local dipole defined via the BC model is an approximation of the local dipole introduced in Ref. (7) via maximally localized Wannier functions. The latter includes full (nonlinear) environmental dependence of the local dipoles, which is computationally significantly more expensive than the simple BC model. So in the current study, we use the BC model, to accelerate the simulation of domain wall dynamics. We also remark that the linear approximation is sufficiently accurate for MD simulations at room temperature, which is sufficiently lower than the ferroelectric phase transition temperature of $PbTiO_3$.

B. MD simulations. With the BC model, electric field-driven (non-equilibrium) MD can be simulated from the evolution under a Hamiltonian H obtained by adding the interaction of the dipoles with the external field E to the Hamiltonian H_0 in the absence of the field, i.e., $H = H_0 - \sum_j \boldsymbol{E} \cdot \boldsymbol{p_j}$. We use LAMMPS (19) and PLUMED (20) for these simulations.

In our MD simulations, we use a $20 \times 40 \times 20$ supercell of $PbTiO_3$. The cell tensor is diagonal and kept fixed along the x, y direction (lattice constant $a = b = 3.91$Å) to mimic the experimental lattice constants in the plane, imposed by the substrate on epitaxially grown thin films. So the area of the 20×40 xy plane of the supercell is fixed as $122.305\mathrm{nm}^2$. This arrangement mimics $PbTiO_3$ thin films deposited on $SrRuO_3/SrTiO_3$ substrates (21–24). We approximate the environmental noise by applying a Langevin thermostat to the thin atomic layers highlighted in pink in Fig.1 of the main text, which contain atoms far away from the initial locations of the domain walls. A barostat is applied in the z direction only. The target pressure $P_z = 2.8 \times 10^4$ bar is determined by roughly matching the calculated average lattice constant c to the experimental value under room temperature and atmospheric pressure. A larger than atmospheric pressure reduces the super-tetragonality error of the adopted DFT functional approximation (see Ref. (7, 16) for more details). This setup approximates a thin $PbTiO_3$ film between conducting electrodes. Notice, however, that the adoption of periodic boundary conditions rules out a precise connection between our model and experimental ultra-thin $PbTiO_3$ samples, where depolarization effects play an important role (25). Another difference between our model and experiment is that the finite size of the supercell along the x/z directions reduces the nucleation-driven growth of the domain (26).

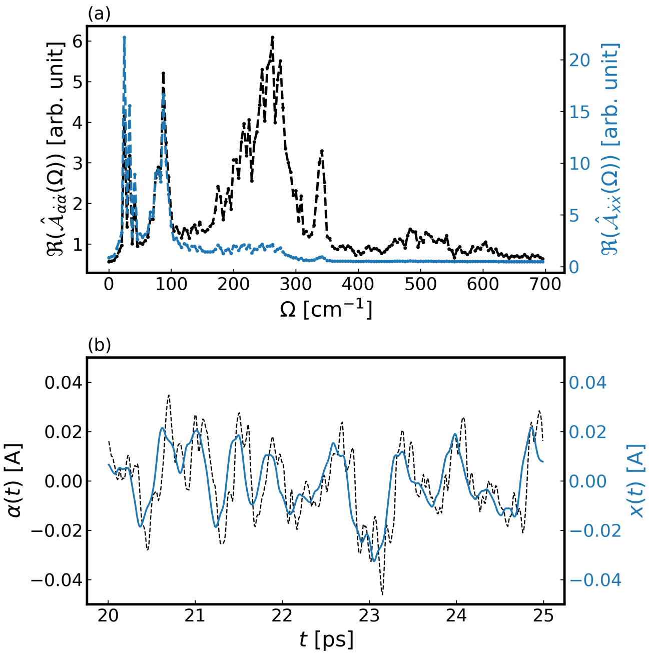

Fig. S3. (a) The real parts of $\hat{\mathcal{A}}_{\dot\alpha\dot\alpha}(\Omega)$ and $\hat{\mathcal{A}}_{\dot x\dot x}(\Omega)$. (b) A segment of a MD trajectory of α (black) under zero external field. The corresponding trajectory of x (blue) in the same time interval is plotted as the blue line for comparison.

C. Temporal coarse-graining. The velocity autocorrelation function (ACF) of the CV α is $\mathcal{A}_{\dot\alpha\dot\alpha}(\tau) = \langle\dot\alpha(\tau)\dot\alpha(0)\rangle$. We use $\hat{\mathcal{A}}_{\dot\alpha\dot\alpha}(\Omega)$ to denote its Fourier transform. Similarly, we use $\hat{\mathcal{A}}_{\dot x\dot x}(\Omega)$ to denote the Fourier transform of the velocity ACF of the temporally coarse-grained CV x. The real parts of $\hat{\mathcal{A}}_{\dot\alpha\dot\alpha}(\Omega)$ and $\hat{\mathcal{A}}_{\dot x\dot x}(\Omega)$ are plotted in Fig. S3(a). Through the temporal coarse-graining from α to x, we see the damped modes of α peaked near 280cm^{-1}, 340cm^{-1}, 500cm^{-1} and 600cm^{-1} are suppressed. Among all, the mode near 280cm^{-1} is not completely eliminated but becomes a continuous spectrum between 100cm^{-1} and 400cm^{-1}. The magnitude of the continuous spectrum is, however, negligible compared to the slow modes between 0cm^{-1} and 100cm^{-1}. So the integration time step of AIGLE with respect to x is mainly limited by the mode peaked near 90cm^{-1}.

To have a more straightforward view of temporal coarse-graining, we plot $\alpha(t)$ and $x(t)$ for the same segment of MD trajectory (no domain motion event) simulated under zero external electric field, in Fig. S3(b). It is clear the fast and small oscillation of α is reduced in x, while the slow and strong oscillation roughly between $0.02A$ and $-0.02A$ is preserved.

D. Training of AIGLE. In the first two training steps of the AIGLE model, the dataset consists of several 400ps long trajectories of x under $E = 2\text{mV/Å}$. The underlying Hamiltonian dynamics is at metastable equilibrium — the twin domain walls are trapped by the periodic potential around a local minimum within the simulated time scale. Under such circumstance, the potential energy surface of the CV is not fully explored, but the fast atomistic degrees of freedom are sufficiently ergodic for determining K and the GAR model. The GAR model contains a feed-forward neural network with two hidden layers (size=10). The $\sigma_{(n)}$ is parameterized by one scalar variable. We use the Adam optimizer with an initial learning rate of 0.01 and an exponential decay rate of 0.9 every 500 steps. We let $n^{\text{GD}} = 10$ and $\epsilon = 0.01$. Convergence is reached within 5000 iterations. The entire dataset is used for every iteration. After training, the colored noise R and the white noise w are both distributed normally on the dataset with negligible off-centering. Non-stationarity is not detected numerically for the GAR model.

In the out-of-equilibrium regime, we fix the memory kernel and the GAR model. Then we retrain the force field $\mathcal{F}(x) = -\partial_x U(x) + pEx$ with MD trajectories (about 1.2ns long in total) simulated independently under different $E \in [2.0, 2.4]\text{mV/A}$. In this dataset, MD can be out-of-equilibrium by creeping down metastable states. Although the entire landscape is explored, the creeping events are too sparse in the dataset for fitting the barrier height U_b accurately. So we predetermine it using metadynamics, a method for computing free energy differences (27), under $E = 0$. The resulting free energy barrier ΔE in energy units is converted to $U_b = \frac{\Delta E a^2}{m}$, where $a = 3.91\text{Å}$ is the lattice constant. All the other parameters in $\mathcal{F}(x)$ are then trained directly on the MD dataset. We use the Adam optimizer with the same setting. Convergence is reached within 5000 iterations. The entire dataset is used at each iteration.

The productive AIGLE model is trained through the steps described above. Next, we examine its agreement with the statistics of MD data. We first extract the reference noise from the MD data with our AIGLE model. Then we use the GAR model to generate the simulated noise time series for comparison. In Fig. S4(a), we report the normalized autocorrelation function (NACF) of the reference noise extracted from the MD data at metastable equilibrium, defined as $C^{\text{MD}}_{RR}(\tau) = \langle R(t_0+\tau)R(t_0)\rangle/\langle R(t_0)^2\rangle$, and the NACF of the corresponding reference residual noise $C^{\text{MD}}_{ww}(\tau) = \langle w(t_0+\tau)w(t_0)\rangle/\langle w(t_0)^2\rangle$, which is also extracted

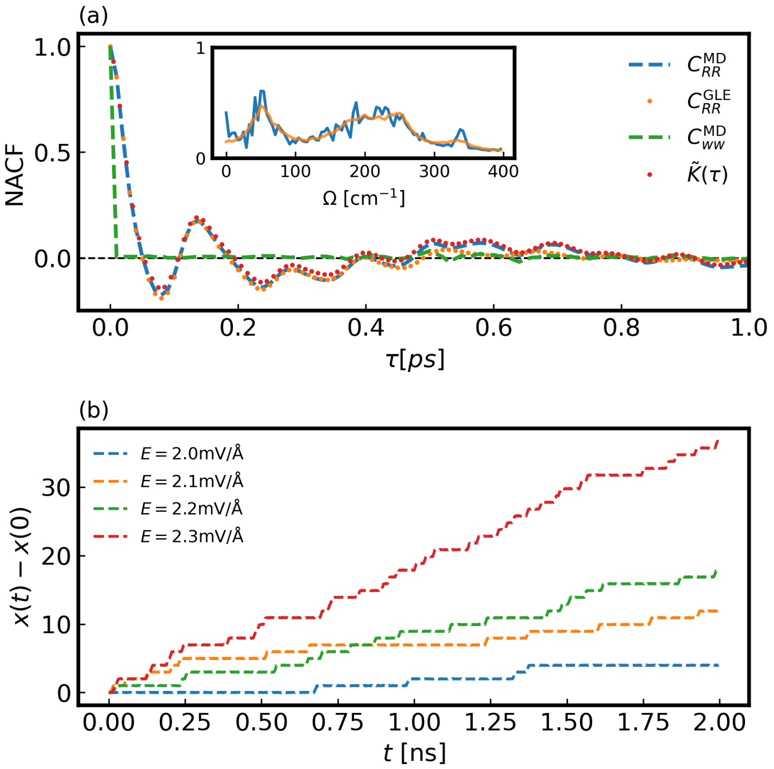

Fig. S4. (a) The NACF of the noise and the rescaled memory kernel $\tilde{K}(\tau)$ for $\tau < 1$ps. For $\tau > 1$ps correlations exhibit a weakly oscillatory pattern similar to that shown here in the vicinity of $\tau = 1$ps. The inset shows $\Re(\hat{C}_{RR}^{\mathrm{MD}}(\Omega))$ (blue) and $\Re(\hat{C}_{RR}^{\mathrm{GLE}}(\Omega))$ (orange) in arbitrary units. $\Re(\hat{C}_{RR}^{\mathrm{MD}}(\Omega))$ displays unphysical oscillations originating from statistical errors due to limited MD data. The first peak in $\Re(\hat{C}_{RR}^{\mathrm{GLE}}(\Omega))$ is located near $50\mathrm{cm}^{-1}$. (b) Time evolution of the CV x under different driving fields E. The waiting time distribution for the domain motion has a long tail.

from the MD data. It is apparent that the cutoff m_A is large enough since $C_{RR}^{\mathrm{MD}}(\tau > m_A\Delta t) \ll 1$. Moreover, $C_{ww}^{\mathrm{MD}}(\tau)$ is delta-like, except for a very small residual correlation for $\tau > m_A\Delta t$. Additionally, both R and w display zero-centered normal distributions on the dataset, indicating that the GAR model successfully reduces the correlated noise in MD trajectories into Gaussian white noise. The NACF of the simulated noise (generated by the GAR model), denoted by $C_{RR}^{\mathrm{GLE}}(\tau)$, is also plotted in Fig. S4(a). $C_{RR}^{\mathrm{GLE}}(\tau)$ captures accurately the dominant oscillatory feature of $C_{RR}^{\mathrm{MD}}(\tau)$, but for a small discrepancy for $\tau > m_A\Delta t$, which is caused by the residual correlation not eliminated by GAR. To display this effect in another way, we compare the Fourier transform of $C_{RR}^{\mathrm{MD}}(\tau)$ and of $C_{RR}^{\mathrm{GLE}}(\tau)$, denoted by $\hat{C}_{RR}^{\mathrm{MD}}(\Omega)$ and by $\hat{C}_{RR}^{\mathrm{GLE}}(\Omega)$, respectively. The corresponding real parts, $\Re(\hat{C}_{RR}^{\mathrm{MD}}(\Omega))$ and $\Re(\hat{C}_{RR}^{\mathrm{GLE}}(\Omega))$, shown in the inset of Fig. S4(a), display overdamped modes within $[0, 400]\mathrm{cm}^{-1}$. As expected, the noise captures mainly broad overdamped modes, due to the interactions of the CV with a bath of fast variables. The main difference between $\Re(\hat{C}_{RR}^{\mathrm{MD}}(\Omega))$ and $\Re(\hat{C}_{RR}^{\mathrm{GLE}}(\Omega))$ occurs for frequencies close to zero, associated to long-time correlation of the noise that can not be captured accurately by a GAR model with finite memory. Given that long-time correlations appear to be weak and fluctuating in $C_{RR}^{\mathrm{MD}}(\tau)$, the errors due to neglecting them in the GAR model should have a negligible overall impact. Finally, in Fig. S4(a), the optimized memory kernel K is rescaled as in $\tilde{K}(\tau) = K(\tau)\frac{C_{RR}^{\mathrm{MD}}(\Delta t/2)}{K(\Delta t/2)}$ to facilitate direct comparison with $C_{RR}^{\mathrm{MD}}(\tau)$. The excellent agreement observed in the figure indicates that the 2FDT is well-satisfied by AIGLE at metastable equilibrium.

The productive AIGLE model is able to simualte efficiently the dynamics of the CV under finite electric field E. Segments of CV trajectories simualted by AIGLE are plotted in Fig. S4(b), where one can see that the time scale of domain displacement events increases from picoseconds to nanoseconds with a modest decrement of E.

E. Ferroelectric switching. The switching process of a ferroelectric thin film typically involves three stages. The first stage is the nucleation of opposite domains at particular nucleation sites. The second stage is the forward growth of the nucleus across the thin film. The last stage is the sideways growth (widening) of the domain. For perovskite oxides, the first two stages are usually much faster than the last one (29). Hence, as a rough approximation, one can consider just the sideways growth of a cylindrical domain for computing the hysteresis loop of a thin film of $\mathrm{PbTiO_3}$. Moreover, when the radius of the switched area is much larger than several nanometers, the curvature dependence of the domain wall velocity should be negligible.

Here, we consider a thin $\mathrm{PbTiO_3}$ film of radius $l \in [1, 1000]\mu$m, initially polarized downward, as shown in Fig. S5(a). An upward square electric field pulse of magnitude E and duration t_p is applied to the film, causing the growth of a central cylindrical domain with upward polarization separated by an interface from the surrounding medium with downward polarization.

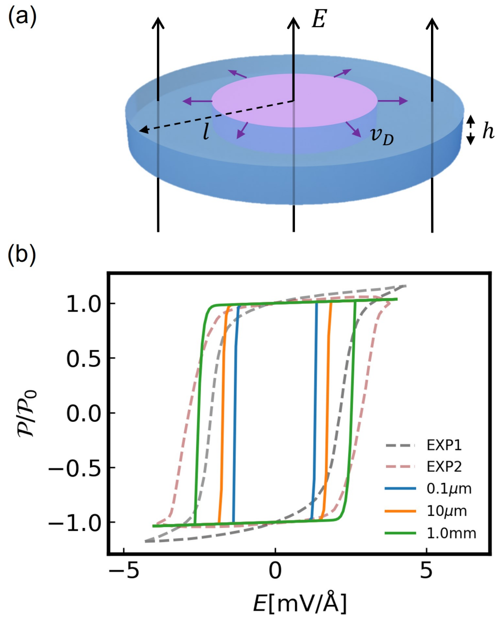

Fig. S5. (a) Schematic representation of the sideways-growth stage of ferroelectric switching. In our model, the effects of the finite film thickness (h) are ignored. The radius of the thin film is l. The purple domain has upward polarization, parallel to the external field E. The blue domain has downward polarization. The speed of growth v_D can be approximated by the speed of a flat domain wall under the same external field, when the upward domain is sufficiently large. (b) The hysteresis loop $\mathcal{P}$, scaled by $1/\mathcal{P}_0$, for $t_p = 1/f = 100\mu$s and different l. $\mathcal{P}_0$ is the remnant polarization at $E = 0$. The "EXP1" experimental data (22) were obtained for the same t_p and unknown l. The "EXP2" data (28) were obtained for $l \approx 144\mu$m and unknown t_p. The remnant polarization in the two experiments is $94\mu\mathrm{C/cm}^2$ (22) and $96\mu\mathrm{C/cm}^2$ (28), respectively. Our atomistic model gives $\mathcal{P}_0 = 72\mu\mathrm{C/cm}^2$.

Alternatively, one could also consider the growing domain to be cuboidal instead of cylindrical. In our atomistic simulations, we find a cuboidal domain, with round corners, is structurally more stable than its cylindrical counterpart when growing (not true when it is shrinking). In this case, approximating v_D with the one computed from an infinite planar domain wall will only be more appropriate.

Because l is large enough, the domain wall velocity can be well approximated by the v_D computed for infinitely large flat domain walls. So after the application of the square pulse, the radius of the upward domain is $r_\uparrow = \min(v_D(E)t_p, l)$. The cross-sectional area is then $A_\uparrow = \pi r_\uparrow^2$ for the upward domain, and $A_\downarrow = \pi l^2 - \pi r_\uparrow^2$ for the downward domain. The lower branch of the hysteresis loop $\mathcal{P}(E)$ can be approximated by the average polarization of the two domains:

$$\mathcal{P}(E) = \frac{A_\uparrow}{\pi l^2}(\mathcal{P}_0 + \chi E) + \frac{A_\downarrow}{\pi l^2}(-\mathcal{P}_0 + \chi E), \qquad [26]$$

where the remnant polarization $\mathcal{P}_0 = 72\mu\mathrm{C/cm}^2$ and the susceptibility $\chi = 0.643\mathrm{nC/mV}$ are extracted from the MD simulations. The upper branch of the hysteresis loop is computed in a similar way.

With this toy model of ferroelectric switching, the hysteresis loop for three typical domain radii l are reported in Fig. S5(b), where we also display the hysteresis loops observed in two experiments (22, 28). The vertical span of the loops is determined by the remnant polarization $\mathcal{P}_0$, which is equal to $72\mu\mathrm{C/cm}^2$ in our atomistic model and is equal to $94\mu\mathrm{C/cm}^2$ and $96\mu\mathrm{C/cm}^2$, respectively, in the two experiments. The loop width depends on the radius l of the film, on the velocity $v_D(E)$ of the interface, and on the coercive field associated to the polarization switch. Quite remarkably, simulations without any empirical input are able to reproduce closely the widths of the loops observed in experiments. The main difference between theory and experiment is that the polarization switch is much sharper in the former than in the latter. Interestingly, had we used Merz's law instead of AIGLE to compute $v_D(E)$, the theoretical loops would have been even sharper, albeit marginally so. The effect is small because near the coercive field, the difference between AIGLE and Merz's law is minor. The discrepancy between theory and experiment in the switching rate of the polarization with E suggests that effects beyond our simple model play a role. Realistic models should describe polarization pinning by point defects (30), the finite thickness of the experimental samples (25, 31), edge effects, the curvature dependence of the domain wall velocity, the morphology of the samples where several polarization domains may be present and eventually coalesce, etc.

5. Details of modeling coarse-grained lattice dynamics

A. DFT-based atomistic models and MD simulations. For this part of the study, we used the same DP model as the one introduced in Sec. A. But for computing the local dipole moments as CVs, we use the Deep Dipole model instead of the effective

Born charge model. The latter is understood as a linear approximation to the former. The Deep Dipole model used here is exactly the same as the one introduced and analyzed in detail, in Ref. (7) via maximally localized Wannier functions.

We carry out two types of MD simulations. The first is $E = 0$ NVT-MD simulation of a 8nm $\times$ 8nm $\times$ 5nm ($20 \times 20 \times 12$ elementary cells) supercell within a single ferroelectric domain. We collect 30ps-long trajectories of all local dipole moments as training data. The second type of MD simulations is electric field-driven NVT-MD simulation of a 20nm $\times$ 20nm $\times$ 5nm ($50 \times 50 \times 12$ elementary cells) supercell initialized with two opposite ferroelectric domains. For $E = 0.5$mV/A and $E = 1$mV/A, we collect respectively 20ps-long trajectories of all local dipole moments as training data. In addition, we collect 200ps-long trajectories of local dipole moments as validation data, used in Fig.3(d,e) of the main text for direct comparison with AIGLE.

B. Training of AIGLE. We first determine the mass of the local dipole moment from the trajectory data $\{\boldsymbol{x}_{(n)}\}$ with the equipartition theorem. We train the parameters in the free energy G through a simple force matching scheme: minimizing the mean-squared-error loss $\mathcal{L}^{\mathrm{MSE}} = \sum_n \|M\boldsymbol{a}_{(n)} + \nabla_{\boldsymbol{x}} G(\boldsymbol{x}_{(n)}) - pM\boldsymbol{E}_{(n)}\|^2$ over the trajectory data collected under zero external field ($E = 0$). Then, we calculate the memory kernel K^* through Eq. (20), followed by the calculation of the Yule-Walker coefficients in Eq. (21). Note that here we do not use any iterative scheme since the free energy is predetermined. Next, with the free energy model and the memory kernel, we extract the noise from the data through $\boldsymbol{R}_{(n)} = M\boldsymbol{a}_{(n)} - \boldsymbol{\mathcal{F}}_{(n)} - \sum_{l=0}^{n-1} MK_{(l+\frac{1}{2})}\boldsymbol{v}_{(n-l-\frac{1}{2})}\Delta t$. We train the GAR model, with $\boldsymbol{R}_{(n)}$ as the time series data and the Yule-Walker coefficients frozen, through the maximum likelihood loss Eq. (24). After the training of the GAR model, we reinforce the 2FDT by modifying the memory kernel from K^* to K_*, as given by Eq. (25). In the end, we retrain the parameters of the free energy and the response coefficient p by minimizing the loss

$$\mathcal{L}^{\mathrm{MSE}'} = \sum_n \frac{\sum_i \|\Delta F_{i,(n)}\|^2 \Theta(|x_{i,(n)}| - x_0)}{\sum_i \Theta(|x_{i,(n)}| - x_0)} + \frac{\sum_i \|\Delta F_{i,(n)}\|^2 \Theta(x_0 - |x_{i,(n)}|)}{\sum_i \Theta(x_0 - |x_{i,(n)}|)}, \qquad [27]$$

where $\Delta \boldsymbol{F}_{(n)} = M\boldsymbol{a}_{(n)} + \nabla_{\boldsymbol{x}} G(\boldsymbol{x}_{(n)}) - pM\boldsymbol{E}_{(n)}$, over the trajectory data collected under $E = 0$, $E = 0.5$mV/A and $E = 1$mV/A. $\Theta(|x_{i,(n)}| - x_0)$ is the Heaviside step function that vanishes if the magnitude of the local dipole moment $x_{i,(n)}$ is smaller than a threshold value $x_0 = 2$eÅ, which distinguishes a local dipole at the interface of opposite domains from the local dipoles in the bulk. The goal is to improve the accuracy of the force field at the interface since these configurations are not included in the first force-matching step. After all these steps, we obtain the AIGLE model yielding the results displayed in the main text.

References

1. SA Adelman, JD Doll, Generalized langevin equation approach for atom/solid-surface scattering: Collinear atom/harmonic chain model. *J. Chem. Phys.* **61**, 4242–4245 (1974).
2. A Paszke, et al., Pytorch: An imperative style, high-performance deep learning library. *Adv. neural information processing systems* **32** (2019).
3. S Theodoridis, *Machine learning: a Bayesian and optimization perspective.* (Academic press), (2015).
4. L Zhang, J Han, H Wang, R Car, W E, Deep potential molecular dynamics: A scalable model with the accuracy of quantum mechanics. *Phys. Rev. Lett.* **120**, 143001 (2018).
5. L Zhang, et al., End-to-end symmetry preserving inter-atomic potential energy model for finite and extended systems. *Adv. Neural Inf. Process. Syst.* **31** (2018).
6. L Zhang, et al., Deep neural network for the dielectric response of insulators. *Phys. Rev. B* **102**, 041121 (2020).
7. P Xie, Y Chen, W E, R Car, Ab initio multi-scale modeling of ferroelectrics: The case of pbtio3. *arXiv preprint arXiv:2205.11839* (2022).
8. J Sun, A Ruzsinszky, JP Perdew, Strongly constrained and appropriately normed semilocal density functional. *Phys. Rev. Lett.* **115**, 036402 (2015).
9. L Zhang, DY Lin, H Wang, R Car, W E, Active learning of uniformly accurate interatomic potentials for materials simulation. *Phys. Rev. Mater.* **3**, 023804 (2019).
10. Y Zhang, et al., Dp-gen: A concurrent learning platform for the generation of reliable deep learning based potential energy models. *Comput. Phys. Commun.* **253**, 107206 (2020).
11. P Giannozzi, et al., Quantum espresso: a modular and open-source software project for quantum simulations of materials. *J. Phys. Condens. Matter.* **21**, 395502 (2009).
12. DR Hamann, M Schlüter, C Chiang, Norm-conserving pseudopotentials. *Phys. Rev. Lett.* **43**, 1494–1497 (1979).
13. G Pizzi, et al., Wannier90 as a community code: new features and applications. *J. Phys. Condens. Matter.* **32**, 165902 (2020).
14. R Resta, D Vanderbilt, *Theory of Polarization: A Modern Approach.* (Springer Berlin Heidelberg, Berlin, Heidelberg), pp. 31–68 (2007).
15. UV Waghmare, KM Rabe, Ab initio statistical mechanics of the ferroelectric phase transition in $pbtio_3$. *Phys. Rev. B* **55**, 6161–6173 (1997).
16. W Zhong, D Vanderbilt, K Rabe, First-principles theory of ferroelectric phase transitions for perovskites: The case of batio 3. *Phys. Rev. B* **52**, 6301 (1995).
17. B Meyer, D Vanderbilt, Ab initio study of ferroelectric domain walls in $pbtio_3$. *Phys. Rev. B* **65**, 104111 (2002).
18. RK Behera, et al., Structure and energetics of 180 domain walls in pbtio3 by density functional theory. *J. Phys. Condens. Matter.* **23**, 175902 (2011).

19. AP Thompson, et al., LAMMPS - a flexible simulation tool for particle-based materials modeling at the atomic, meso, and continuum scales. *Comp. Phys. Comm.* **271**, 108171 (2022).
20. GA Tribello, M Bonomi, D Branduardi, C Camilloni, G Bussi, Plumed 2: New feathers for an old bird. *Comput. Phys. Commun.* **185**, 604–613 (2014).
21. T Morita, Y Cho, Epitaxial pbtio3 thin films on srtio3 (100) and srruo3/srtio3 (100) substrates deposited by a hydrothermal method. *Jpn. J. Appl. Phys.* **43**, 6535 (2004).
22. WH Kim, SM Yoon, JY Son, Ferroelectric domain wall motion in epitaxial pbtio3 and bifeo3 thin films. *Mater. Lett.* **124**, 47–49 (2014).
23. R Nishino, TC Fujita, F Kagawa, M Kawasaki, Evolution of ferroelectricity in ultrathin pbtio3 films as revealed by electric double layer gating. *Sci. reports* **10**, 1–8 (2020).
24. Ø Dahl, JK Grepstad, T Tybell, Polarization direction and stability in ferroelectric lead titanate thin films. *J. Appl. Phys.* **106**, 084104 (2009).
25. M Dawber, P Chandra, P Littlewood, J Scott, Depolarization corrections to the coercive field in thin-film ferroelectrics. *J. Phys. Condens. Matter.* **15**, L393 (2003).
26. YH Shin, I Grinberg, IW Chen, AM Rappe, Nucleation and growth mechanism of ferroelectric domain-wall motion. *Nature* **449**, 881–884 (2007).
27. A Barducci, G Bussi, M Parrinello, Well-tempered metadynamics: A smoothly converging and tunable free-energy method. *Phys. Rev. Lett.* **100**, 020603 (2008).
28. T Morita, Y Cho, Epitaxial PbTiO3thin films on SrTiO3(100) and SrRuO3/SrTiO3(100) substrates deposited by a hydrothermal method. *Jpn. J. Appl. Phys.* **43**, 6535–6538 (2004).
29. M Dawber, KM Rabe, JF Scott, Physics of thin-film ferroelectric oxides. *Rev. Mod. Phys.* **77**, 1083–1130 (2005).
30. L He, D Vanderbilt, First-principles study of oxygen-vacancy pinning of domain walls in pbtio 3. *Phys. Rev. B* **68**, 134103 (2003).
31. V Nagarajan, et al., Thickness dependence of structural and electrical properties in epitaxial lead zirconate titanate films. *J. Appl. Phys.* **86**, 595–602 (1999).
